# Ecology, genetic diversity, and population structure among commercial varieties and local landraces of *Capsicum* spp. grown in northeastern states of India

**DOI:** 10.3389/fpls.2024.1379637

**Published:** 2024-04-04

**Authors:** Veerendra Kumar Verma, Avinash Pandey, Anbazhagan Thirugnanavel, Heiplanmi Rymbai, Niharika Dutta, Amit Kumar, Tshering Lhamu Bhutia, Anjani Kumar Jha, Vinay Kumar Mishra

**Affiliations:** ^1^ Division of System Research & Engineering, ICAR Research Complex for North Eastern Hill Region, Umiam, Meghalaya, India; ^2^ School of Genomics and Molecular Breeding, ICAR-Indian Institute of Agricultural Biotechnology, Ranchi, Jharkhand, India; ^3^ ICAR-Central Citrus Research Institute, Nagpur, Maharashtra, India; ^4^ ICAR Research Complex for North Eastern Hill Region, Gangtok, Sikkim, India; ^5^ ICAR-Indian Institute of Horticultural Research, Bengaluru, India

**Keywords:** chili peppers, geographical indication, ecology, diversity, molecular marker

## Abstract

Northeastern states of India are known for unique landraces of *Capsicum* spp. with geographical indications. However, little information is available about these valuable landraces of chillies. Surveys and collections were carried out in niche areas to find out their ecology and diversity through morphological traits and molecular analysis using microsatellite markers. Our result characterized the ecology of niche areas as cool (11.0°C–20.7°C) and humid (>60% relative humidity) climates for dalle-chilli (*Capsicum annuum* L.); mild-warm (12.2°C–28.6°C) and humid for king-chilli (*C. chinense* Jacq.); and cool to warm (11.3°C–33.1°C) and humid for bird’s eye chilli (*C. frutescens* L.) during the crop period. The canonical correspondence analysis has shown the significant impact of temperature on the agro-morphological traits and distribution of the landraces in their niche areas. A wide variability was observed for different quantitative traits and yield attributing characters (fruit length, diameter, weight, and yield), showing high heritability (97.0%–99.0%), and genetic advance as a percentage of the mean (119.8%–434.0%). A total of 47 SSR markers used for the molecular analysis generated 230 alleles, ranging from 2 (HPMSE-7) to 10 (HPMSE-5), with an average of 4.89 alleles per locus. The average polymorphism information content was also high (0.61) and ranged from 0.20 (HPMSE-7) to 0.85 (CAMS-91). The observed average heterozygosity was lower than the expected value. Analysis of molecular variance has shown significant variation within (69%) and between (31%) of the populations of *Capsicum* spp. Based on Nei’s genetic distance, bird’s eye chilli and king-chilli were found to be closer to each other, whereas dalle-chilli, a tretraploid species, was closer to hot pepper (*C. annuum*). However, the flower size of dalle-chilli was large and found closer to king-chilli in color and differs from *C. chinense* due to the presence of calyx teeth. For quality traits, landraces king-chilli, dalle-chilli, and bird’s eye chilli have shown 2.8, 2.0, and 1.4 times higher average capsaicin and 0.46, 0.25, and 0.22 times higher average oleoresin content over the hot pepper, respectively. The knowledge of ecology and diversity can be used in identifying new areas for production, selection of elite lines, conservation, and crop improvement.

## 1 Introduction

*Capsicum* spp. is the most widely grown solanaceous crop. In worldwide view, the immature fruits, mature red, yellow to purplish, are consumed as vegetables, and dried red ripe fruits are used as a spice. The food industry is the primary user of chillies, where they are used as coloring and flavoring agents in processed products, snacks, candies, soft drinks, and alcoholic beverages (Pino et al., 2007). Oleoresin, a value-added product of *Capsicum* spp., is used as a food additive for its color and pungency. It is a resin-like viscous material that represents the complete flavor and non-volatile resinous fraction present in the spices. The resinous fraction comprises heat components (capsaicinoids), fixatives, natural antioxidants, and pigments (Sharma and Sharma, 2004). India is a major supplier of spice oil and oleoresin to global markets and earned ≈ 544.42 million (USD) from the export of 21,921 metric tons in 2021–2022 (Spice Board India., 2023). Spice oil and oleoresin account for 16% of the export share of spices in India. Capsaicinoids, a group of active compounds, are responsible for the pungency of the fruits. Among them, capsaicin is the most common capsaicinoid, has several nutraceutical and medicinal properties, and is used to relieve the pain of peripheral neuropathy (Backonja et al., 2008) in topical ointments, nasal sprays, and dermal patches, typically in concentrations between 0.025% and 0.25%. It also possesses antibacterial properties (Dorantes et al., 2000; Koffi-Nevry et al., 2012; Nascimento et al., 2014; El Ksibi et al., 2015) as well as anti-inflammatory and antioxidant activities and can inhibit various cancer cells (Mori et al., 2006; Baek et al., 2008). Furthermore, capsaicin inhibits obesity by decreasing energy intake (Reinbach et al., 2009), adipose tissue weight, and serum triglyceride through stimulation of lipid mobilization (Kawada et al., 1986).

Among the *Capsicum* spp., hot pepper is known to grow most widely under diverse climatic conditions in India. In North Eastern (NE) India, it is grown as a pure crop under irrigated conditions after the harvesting of rice in the valleys and as a rainfed crop under a mixed cropping system in the Jhum/Shifting cultivation. Moreover, the cropping period of the chillies varied in the region due to the diverse climate. Generally, the sowing period is during mid-February; however, it is January in Mizoram and Barak valleys due to mild winter and February to March in Jhum/shifting cultivation areas in the mid-hills. Out of the 30 species reported in the genus *Capsicum*, five species (*C. annuum* L., *C. frutescens* L., *C. chinense* Jacq., *C. baccatum* L., and *C. pubescens* R. and P.) are popular among them (Bosland and Votava, 2000). Previously, *C. annuum* (hot pepper and sweet pepper) was mainly grown for its fresh consumption, cooking, and processed products, like a dry powder for color and pungency in cooking (Selvakumar et al., 2022). Over a while, the other species of *Capsicum* have also gained importance, mainly due to their high oleoresin and capsaicin content, in the food and pharmaceutical industries in both domestic and international markets. The NE region of India comprises eight states with diverse agro-ecological conditions and is considered one of the richest reservoirs of flora and fauna as part of the Indo-Myanmar biodiversity hotspot, one of the 36 biodiversity hotspots recognized around the world (Myers et al., 2000). In the region, besides commercial cultivars of hot and sweet pepper (*C. annuum*), there are popular landraces, i.e., king-chilli/bhut jolokia (*C. chinense*), bird’s eye chilli (*C. frutescens*), dalle-khursani chilli/dalle-chilli, and cherry chilli (*C. annuum*) grown widely under different ecology and cropping systems. The capsaicin content of king-chilli and bird’s eye chilli has been reported to be the highest when grown in their niche areas compared to other country places (Tiwari et al., 2005; Verma et al., 2020). Furthermore, king-chilli has been found to be the most economical crop with higher (> 4.0) benefit-cost ratio over other high-value crops such as tomato, capsicum, and cucumber grown under protected conditions (Verma et al., 2018a). Botanically, king-chilli is considered *C. chinense* (Malakar et al., 2019). However, based on molecular analysis, king-chilli has been reported as a natural interspecific hybrid between *C. chinense* and *C. frutescens* (Bosland and Baral, 2007; Purkayastha et al., 2012; Kehie et al., 2016) in their niche areas, and seeds have erratic germination behavior as compared to other species (Verma et al., 2018b). Dalle-Khursani chilli, a winter-hardy, tall, and perennial *Capsicum annuum*, is grown widely in the mid-hills of Sikkim and Darjeeling (West Bengal), Nepal, and Bhutan. Based on agro-morphological traits, this species differs from other *Capsicum* spp (Jha et al., 2017). Furthermore, bird’s eye chilli (*C. frutescens*) is another prevalent species, grown in the entire eastern Himalayas of India. It is mainly grown in backyards and *Jhum* lands (shifting cultivation), and maximum diversity has been reported in Mizoram, Manipur, and Meghalaya (Dutta et al., 2017). Based on the landraces’ diversity, ecology, and quality traits, the geographical indications (GIs) tag has been granted to Nagaland for king-chilli (2008), Sikkim for dalle-chilli (2020), and Mizoram state for bird’s eye chilli (2023) by the government of India. Today, there are different value-added products processed and marketed commercially for these species under a particular brand, such as “Sikkim Supreme” pickles of dalle-chillies.

Diverse genetic resources are essential for crop improvement (Maxted et al., 2008; Maxted and Kell, 2009; Perrino and Wagensommer, 2021; Perrino and Wagensommer, 2022; Accogli et al., 2023). The region, in spite of having potential genetic diversity in the landraces of *Capsicum* spp., is yet to be efficiently utilized. Moreover, there is a need for systematic study on diversity, ecology of the landraces, evolution, and phylogenetic relationships, especially in popular landraces such as king-chilli and dalle-chilli. The genetic characterization of some *Capsicum* spp. has been previously carried out using agro-morphological and quality attributes (Dutta et al., 2017; Vaishnavi et al., 2018), cytogenetics (Jha et al., 2017), and different generations of markers such as sodium dodecyl-sulfate polyacrylamide gel electrophoresis (Mena et al., 2019); randomly amplified polymorphic DNA (RAPD) markers (Bosland and Baral, 2007), simple sequence repeats (SSR) markers (Adluri et al., 2017; Colney et al., 2018; Baruah et al., 2019), and internal transcribed spacer sequence of nuclear ribosomal DNA (Purkayastha et al., 2012); and sequencing of the ribosomal RNA (rRNA) gene-internal transcribed (ITS) region (Kehie et al., 2016). Due to genome-wide coverage, robust and high reproducibility, co-dominant inheritance, high polymorphism with multiple alleles per locus, and transferability between species, SSR markers have been widely used in fingerprinting, analysis of genetic diversity and population structure, association mapping, and linkage mapping (Lee et al., 2004; Minamiyama et al., 2006; Mimura et al., 2012; Yumnam et al., 2012; Sugita et al., 2013; Dhaliwal et al., 2014; Lee et al., 2016; Adluri et al., 2017; Colney et al., 2018; Baruah et al., 2019; Pereira-Dias et al., 2019; Solomon et al., 2019). Keeping the above facts in view, the present investigation was conducted to study the ecology of the GIs tags and other local landraces of *Capsicum* spp.; to study the genetic diversity and phylogenetic relationships in landraces of *Capsicum* spp. grown in the region based on SSR markers; to identify high-yielding genotypes; and to analyze economically important traits such as capsaicin and oleoresin content in superior genotypes.

## 2 Materials and methods

### 2.1 Survey and collection

**Figure 1:**
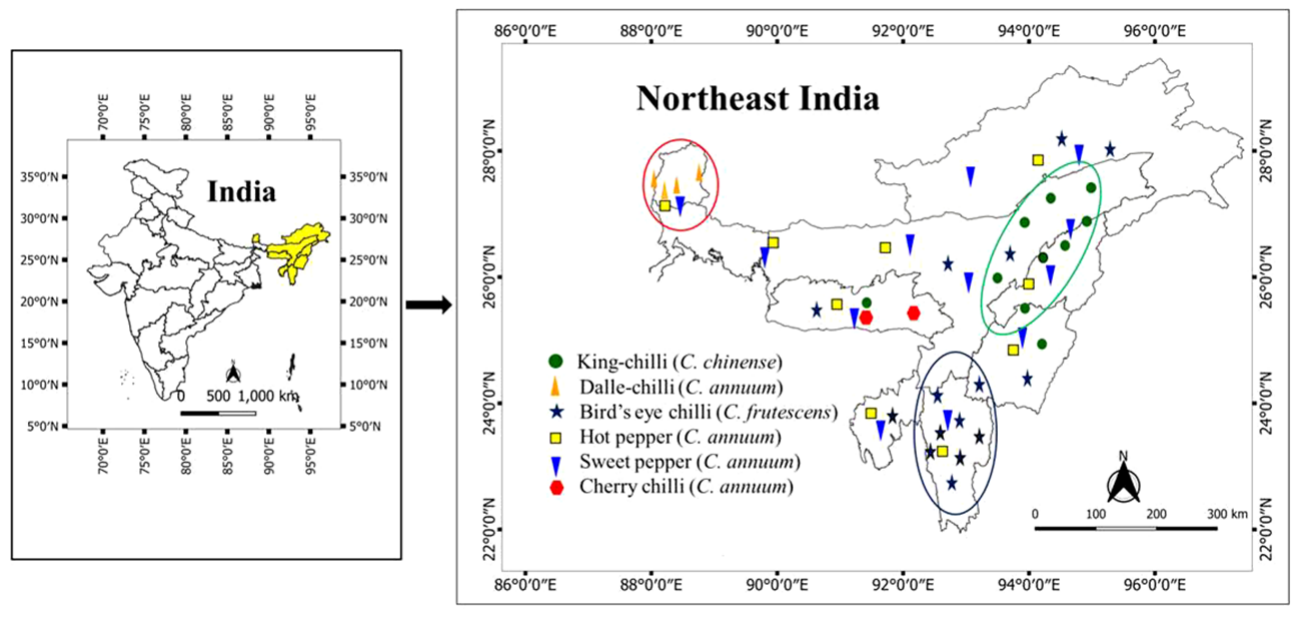
Distribution and niche areas of the popular landraces of *Capsicum* spp. The niche area for dalle-chilli (*Capsicum annuum*) is marked by red circle, king-chilli (*C. chinense*) by green circle, and bird’s eye chillies (*C. frutescens*) by blue circle.

A total of 106 genotypes of *Capsicum* spp., belonging to 68 hot peppers (*C. annuum* L.) including seven commercial cultivars (i.e., Kashi Anmol, Mahalakshmi, Surajmukhi, Japanese Long, Arka Lohit, Pusa Jwala, and Utkal Yellow), six sweet pepper (*C. annuum* L.) cultivars/hybrids [Capsicum Long, Yellow Wonder, Orobelle (F_1_), Nishant, California Wonder, and Capsicum Red Long], and two cherry chillies (*C. annuum* L.), and GI tag landraces including 16 bird’s eye chillies (*C. frutescens* L.), nine dalle-chillies (*C. annuum* L.), and five king-chillies (*C. chinense* Jacq.) were collected (selective) through surveyed from the farmers’ fields and local markets in the different parts of the region, especially from the eco-geographical niche area (**Figure 1**). The period of survey and collection was carried out during July to August, during the peak flowering and fruiting stages. For studying the climate of the niche areas, observations on weather parameters (temperature, rainfall) were taken for different (four to five) growing pockets/locations in each area from regional centers of the ICAR Research Complex for North Eastern Hill Region, located in each state, and also from https://en.climate-data.org (**Table 1**).

**Table 1:** Weather parameters of the major growing pockets of the popular landraces of *Capsicum* spp.

Sl. no.	Name of location	Annual temperature (°C)		Average annual temperature (°C)		Average (monthly) temperature (°C) during crop period		Annual rainfall (mm)	Elevation (m)	Landraces**
1		Min	Max	Min	Max	Min	Max			
2	Darjeeling, West Bengal	3.3	20.2	6.6	17.8	11.7	17.80	2,547	2,000	Dalle-chilli
3	Mirik, West Bengal	6.3	22.6	9.6	20.1	11.0	20.10	2,876	1,495	Dalle-chilli
4	Ravangla, South Sikkim	2.9	21.5	7.0	18.6	12.0	18.6	2,430	1,820	Dalle-chilli
5	Gangtok, Sikkim	5.5	23.7	9.9	20.7	14.7	20.7	2,578	1,650	Dalle-chilli
6	Lunglei, Mizoram	11.0	28.3	16.0	23.5	16.0	23.5	3,480	722	Bird’s eye chilli
7	Kolasib, Mizoram	10.6	28.5	16.3	24.6	16.3	24.6	2,805	888	Bird’s eye chilli
8	Saiha, Mizoram	10.4	28.3	15.5	23.5	15.5	23.5	2,876	1,056	Bird’s eye chilli
9	Hailakandi, Assam	11.0	28.3	16.0	22.8	16.0	22.8	3,480	21	Bird’s eye chilli
10	Nongpoh, Meghalaya	9.2	29.0	15.1	25.9	15.1	25.9	3,200	485	Bird’s eye chilli/cherry chilli
11	Ziro, Arunachal Pradesh	2.6	26.2	9.4	22.2	11.3	22.2	2,005	1,571	Bird’s eye chilli
12	Imphal, Manipur	7.8	29.2	14.5	24.6	14.5	24.6	1,581	786	Bird’s eye chilli/King-chilli
13	Kohima, Nagaland	4.2	25.4	11.2	22.0	12.7	22.0	1,863	1,444	King-chilli
14	Dimapur, Nagaland	9.3	32.3	16.6	28.6	16.6	28.6	1,560	145	King-chilli/Bird’s eye chilli
15	Peren, Nagaland	4.9	26.0	11.7	22.5	13.5	22.5	2,056	1,277	King-chilli
16	Tameglong, Manipur	5.9	25.9	12.2	22.2	12.2	22.2	3,336	1,266	King-chilli
17	Kamrup, Assam	8.0	38.6	12.0	33.1	17.6	33.1	1,600	50.0	Hot pepper/Birds eye chilli
18	–	–	–	–	–	12.3	25.8	1,600	–	Sweet pepper*

*The temperature data for sweet pepper has been taken only for the growing season, as it is grown in entire region during cool season.

**The species of the landraces are *C. annuum* (dalle-chillies), *Capsicum annuum* (cherry chilli, *C. frutescens* (bird’s eye chillies), *C. chinense* (king-chillies) and *C. annuum* (hot pepper and sweet pepper).

### 2.2 Performance evaluation for growth and yield attributes

All the genotypes were evaluated under open field conditions (February to October 2018–2020) for two consecutive years. The experimental site was in Horticulture Farm, ICAR Research Complex for NEH Region, Umiam, Meghalaya (latitude 25°41′N and 92°55′E longitude), located under mid-hills at 960 meter above mean sea level (m a.s.l.). Yearly, rainfall ranged from 2,200 mm to 2,551 mm, and the average minimum and maximum temperatures during the crop period were 18.0°C and 28.3°C, respectively. This location has inceptisol soils of sandy texture and acidic reaction (pH 5.4). The pre-treated seeds with the fungicide Captan were sown in the nursery each year during the first week of January. One-month-old seedlings were transplanted on raised beds (3.5 m ×2.0 m in size, with 28 plants per plot) at 45 cm × 45 cm spacing between line to line and plant to plant, respectively. The genotypes were evaluated in a Randomized Complete Block Design. The package of practices was followed as per recommendations of the Institute in the region. Observations for nine growth and yield attributes, such as average fruit length (cm), fruit diameter (cm), fruit weight (g), leaf length (cm), leaf width (cm), leaf area (cm^2^), number of fruits per plant, yield per plant (g), and number of seeds per fruit, were taken on six plants/fruits in each replication. The observations for flower color were taken as per the Royal Horticultural Society Color Charts (2015).

#### 2.2.1 Data analysis

Broad-sense heritability (%) was estimated as suggested by Allard, (1960). Genetic advance (GA), percentage of the mean (GAM), and assuming selection of the superior 5% of the genotypes were followed according to the methods illustrated by Johnson et al. (1955). The canonical correspondence analysis (CCA) was carried out to study the association between the weather parameters (average minimum, maximum, and differential monthly temperature of the cropping period) of the niche area and the quantitative traits of the genotypes, which was analyzed as described by ter Braak (1986) using the R software package “Vegan” developed by Oksanen et al. (2017).

### 2.3 Quality analysis

Two of the most economically important quality traits, namely, as capsaicin and oleoresin content, were analyzed for the selected superior genotypes of different landraces of the *Capsicum* spp. Capsaicin content in chilli powder from red ripe fruits was estimated spectrophotometrically following the method of Thimmaiah (1999) and oleoresin by Mathew et al. (1971).

### 2.4 Molecular analysis

#### 2.4.1 Plant samples and DNA extraction

The total gDNA was extracted from fresh young leaves (2 g) of 1-month-old seedlings using the modified cetyltrimethyl ammonium bromide method (Saghai-Maroof et al., 1984) with an addition of polyvinylpyrrolidone (1%) in the extraction buffer. The sample was then ground to a fine powder using liquid nitrogen. Nanodrop™ 1000 Spectrophotometer (Thermo Scientific, USA) was used for DNA quantification.

#### 2.4.2 PCR and gel electrophoresis

PCR reactions were carried out in a Thermal Cycler (Veriti, Applied Biosystem, Singapore). Each 20 ml of reaction mixture contained 1X reaction buffer [10 mM Tris-HCl (pH 8.3) and 50 mMKCl), 2.5 mM MgCl_2_, 1 U of Taq DNA polymerase; 200 mM each of dATP, dTTP, dCTP, and dGTP (all reagents from Thermo Fisher Scientific, Lithuania); and 0.6 mM of primer and approximately 25 ng of template DNA. A total of 47 polymorphic expressed sequence tag-Simple Sequence Repeat primers reported earlier in different *Capsicum* spp (Yi et al., 2006). were screened and selected for the analysis. The PCR amplification conditions were as follows: an initial extended step of denaturation at 94°C for 5 min, followed by 30 cycles of denaturation at 94°C for 45 s, primer annealing at 55°C for 45 s, and primer elongation at 72°C for 1 min, followed by an extended elongation step at 72°C for 7 min. Reaction products were mixed with 2 µL of 6× loading dye (Thermo Fisher Scientific, Lithuania) and spun briefly in a microfuge before loading. The amplification products were electrophoresed on a 3.0% agarose gel at 60 V. Gels were stained with ethidium bromide and documented using a Chemidoc™ (Bio-Rad, California, USA).

#### 2.4.3 Data analysis

The molecular weights of bands (amplicons) were estimated using a 50-bp DNA ladder, and the homology of bands (amplicons) was based on the migration distance in the gel. Only reproducible SSR amplicons obtained from each entry were resolved as a band on the gel system, and the data sets were used to calculate the number of alleles (NA), effective number of alleles (Ne), availability (A), Shannon’s information index (I), observed heterozygosity (Ho), expected heterozygosity (He), fixation index (F), major allele frequency (MAF), and the polymorphism information content (PIC) for each locus using Power Marker software. For phylogenetic analysis, neighbor-joining (NJ) dendrogram based on Nei’s genetic distance (Nei, 1983) was generated with 100 bootstrap values using Power Marker software. Nexus NJ bootstrap file, exported from Power Marker software, was used in MEGA 7 software to generate the dendrogram. GenAlEx 6.51b2 software was used for analysis of molecular variance (AMOVA), coefficient of gene differentiation (*Gst*) and confirmation of the Hardy–Weinberg equilibrium (HWE). It was also used to calculate pair-wise Nei’s genetic distance. Population structure analysis was done using STRUCTURE 2.3.4 software (Pritchard et al., 2000). The optimum value of K was determined in the Structure Harvester online software 9 Earl and vonHoldt (2012) (http://taylor0.biology.ucla.edu/structureHarvester/). Principal coordinate analysis (PCoA) based on allele frequency was done using XLSTAT software. To visualize the relationship among groups of the genotypes, multiple correspondence analysis (MCA) was carried out using R software with FactoMineR, factoextra, and ade4 packages (Husson et al., 2013).

## 3 Results

### 3.1 Ecology of the local landraces

**Figure 2:**
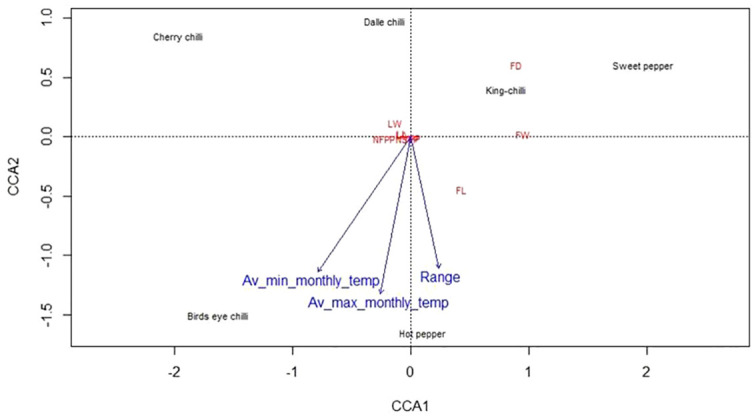
Canonical correspondence analysis (CCA) biplot depicting the relationship between climatic variable (temperature) and landraces of the *Capsicum* spp. based on quantitative traits. The species are *C. annuum* (dalle-chillies), *Capsicum annuum* (cherry chilli), *C. frutescens* (bird’s eye chillies), *C. chinense* (king-chillies), and *C. annuum* (hot pepper and sweet pepper).

The weather parameters presented in **Table 1** show the differences in the climatic conditions of the niche area of the Capsicum landraces grown in the region (**Figure 1**). The ecology of the niche area of dalle-chillies (*C. annuum*) is characterized by a cool and humid climate, with temperatures ranging from minimum (11.0°C–14.7°C) to a maximum (17.8°C–20.7°C) during the crop period (March to October). Likewise, the average minT of the niche area (Nagaland, Assam, and Manipur) of landrace king-chilli (C. chinense) was 12.2°C–16.6°C, and the maxT was 22.2°C–28.6°C during the crop period, which is mildly warmer than the weather parameters of a niche area of dalle-chilli. Furthermore, bird’s eye chillies (*C. frutescens*) were found adoptive to a wider range of temperature 11.3°C to 33.1°C during the crop period. The weather parameters (temp) have shown a significant impact on the variability of the quantitative traits. The first three axis of CCA have explained 45.0%, 9.84%, and 2.85% of the total variance of the landrace–weather parameter relationship, respectively. The axis-1 of CCA biplot (**Figure 2**) has differentiated the large fruited genotypes of sweet pepper (*C. annuum*) and king chillies (*C. chinense*) from small fruited genotypes of bird’s eye chillies (*C. fruitescens*) and cherry chillies (*C. annuum*). However, hot pepper and dalle chilles (*C. annuum*) were found close to the axis. The axis-2 of CCA biplot has differentiated the landraces of bird’s eye chillies (*C. frutescens*) and hot pepper (*C. annuum*) adopted to higher temperatures from the landraces of *C. annuum* (dalle-chillies, cherry-chillies, and sweet pepper) and *C. chinense* (king-chillies) adopted to low and mild temperature conditions, respectively.

### 3.2 Performance of *Capsicum* spp. for growth and yield attributes

**Figure 3:**
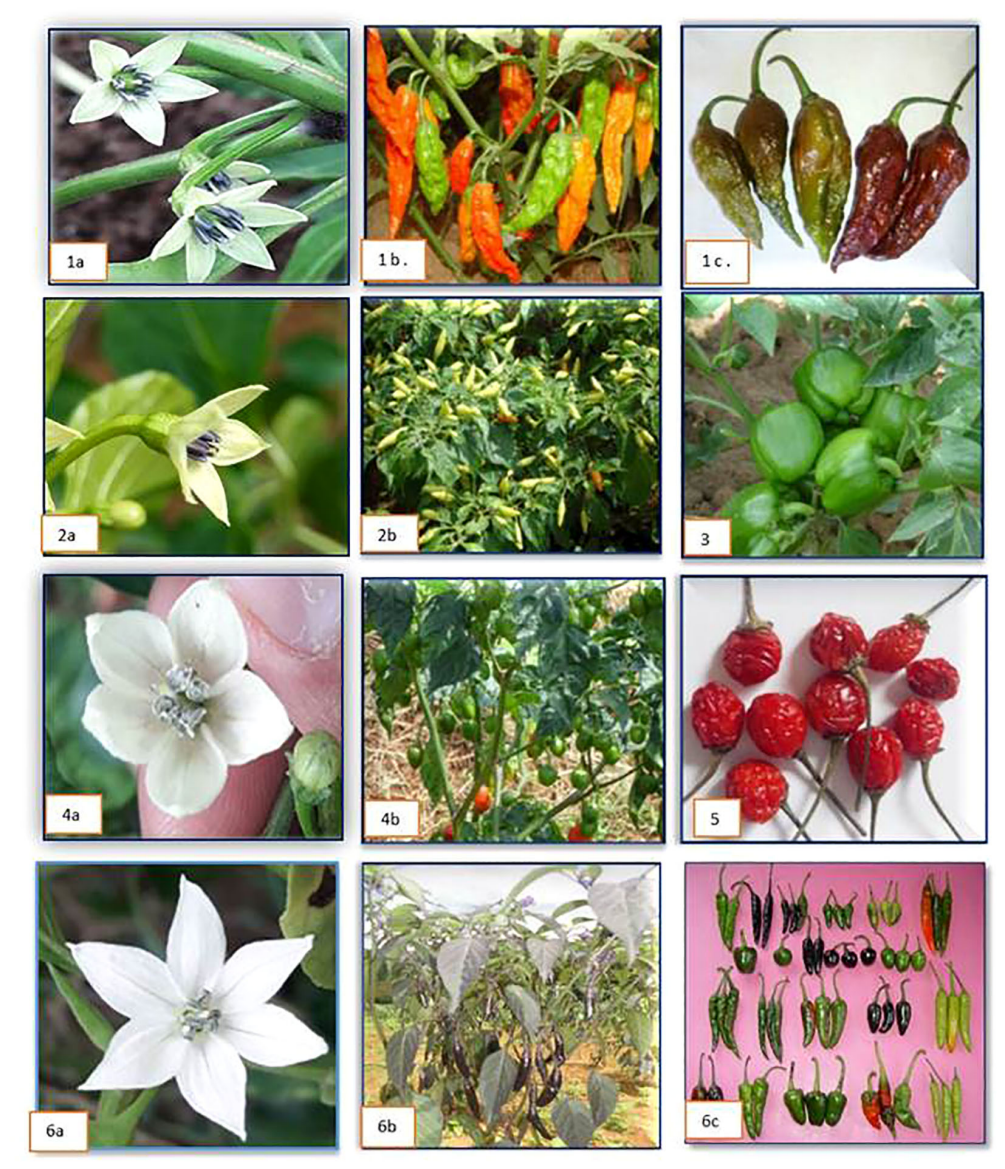
Flower and fruits of different *Capsicum* spp. 1 (a–c) = king-chilli (*Capsicum chinense*); 2 **(a, b)** = bird’s eye chilli (*C. frutescens*); 3 = immature fruit of sweet pepper (*C. annuum*) cv. California Wonder; 4 **(a, b)** = dalle-chilli (*C. annuum*); 5 = cherry chilli (*C. annuum*); 6 (a–c) = flower and different fruit types of hot pepper (*C. annuum*).

The popular landraces of the region were collected from their niche area and evaluated for the agro-morphological traits. A total of nine morphologically distinct genotypes of dalle-chilli (*Capsicum annuum*) landraces differs from the common chillies were collected from Darjeeling (West Bengal) and Sikkim. In *Capsicum* spp., the calyx teeth are considered one of the distinct characters for the differentiation of the species. In our study, we could find that dalle-chilli had a large flower with teeth on the calyx and a light yellow-green corolla (**Figure 3**). Growth and yield attributes showed wide variations; the morphological characters ranged as follows: fruit weight (1.90–3.75 g), fruit length (1.15–2.55 cm), fruit diameter (1.45–1.70 cm), seeds (15.50–62.0 number per pod), leaf area (49.59–149 cm^2^), number of fruits (69.50–113.50), and yield (192.72–422.40 g) per plant. Among the collections, the highest yielding genotypes for red-ripe fruits were SKCC-7 (422.4 g per plant), followed by SKCC-5 and SKCC-1 (**Supplementary Table 1**).

Similarly, five distinct genotypes of king-chilli (*C. chinense*) were collected from Nagaland and Manipur. The corolla of flower was intense yellow-green in color and had a calyx with teeth (**Figure 3**). The fruit weight ranged from 3.65 g to 6.25 g, fruit length (3.25–7.25 cm), fruit diameter (2.45–3.55 cm), seeds number (33.0–39.0 per pod), leaf area (77.19–170.05 cm^2^), fruits (28.0–45.50 number per plant), and yield (118.80–278.30 g per plant). Genotypes such as King-chilli-1 followed by King-chilli-4, and King-chilli-2 had the highest yield for red-ripe fruits (**Supplementary Table 2**). Cherry chillies (*C. annuum*) another landrace is grown primarily in backyards for self-consumption purposes only. Two genotypes of cherry chilli, i.e., MLCC-34 and MLCC-35, collected and evaluated had smaller flower sizes and colors similar to those of *C. annuum*. The highest yield was recorded from the bold fruiting genotype MLCC-34 (115.50 g per plant) (**Supplementary Table 3**).

Among the genotypes (Tiwari et al., 2005) of the bird’s eye chilli (*C. frutescens*), the wider variability was also observed for different growth and yield traits (**Supplementary Table 4**). Flowers were smaller in size, were yellowish green in color, and had teeth on the calyx. Fruit colors were observed as cream, light green, and dark green. The range of growth and yield attributes varied from 0.30 g to 1.95 g for fruit weight, from 0.70 cm to 4.15 cm for fruit length, from 0.35 cm to 1.05 cm fruit diameter, from 5.0 to 29.0 numbers per fruit seed, from 22.70 cm^2^ to 140.25 cm^2^ leaf area, from 50.50 to 135.0 for number of fruits, and 24.75 g to 203.28 g for yield per plant. Among the collections, the high-yielding genotypes for red-ripe fruits yield per plant were MZBEC-13 (203.28 g) followed by MZBEC-1 (108.90 g), and MLCC-1 (95.04 g).

A total of 68 genotypes of hot pepper (*C. annuum*), including seven commercial cultivars, were evaluated and showed wide variations in different traits (**Supplementary Table 5**). Fruit and flower colors were observed as white, pale yellow, and purple with teeth on the calyx. Fruit weight ranged from 1.75 g to 6.75 g; fruit length ranged from 1.25 cm to 13.55 cm; fruit diameter ranged from 0.55 cm to 2.15 cm; seeds number ranged from 11.0 to 109 per fruit; leaf area ranged from 24.34 cm^2^ to 128.46 cm^2^; number of fruits ranged from 22.0 to 167.5; and yield ranged from 60.72 g to 618.75 g per plant. Among the collections, the high-yielding genotypes for red-ripe chillies per plant were MLCC-36 (618.75 g), followed by ASCC-2 (564.3 g), NLCC-4 (423.5 g), NLCC-10 (411.4 g), Kashi Anmol (415.0 g), and TRCC-7 (385.0 g). Likewise, six genotypes of sweet pepper (*C. annuum*) were also evaluated for growth and yield attributes; fruit weight ranged from 3.70 g to 69.00 g; fruit length ranged from 3.60 cm to 8.60 cm; fruit diameter ranged from 1.45 cm to 5.15 cm; seed ranged from 23.50 to 142.50 numbers per fruit; leaf area ranged from 44.58 cm^2^ to 102.59 cm^2^; number of fruits ranged from 9.0 to 40.0; and yield ranged from 175.56 g to 748.00 g per plant. The high-yielding genotypes for mature marketable fruits per plant were identified as California Wonder, followed by Yolo Wonder and Nishant (**Supplementary Table 6**).

### 3.3 Estimation of genetic parameters

The estimation of genetic parameters revealed a wide range of variations for the mean values of all the traits (**Table 2**). The genotypic coefficient of variation (GCV) and phenotypic coefficient of variation (PCV) values ranged from 28.46% to 211.0% for leaf length and 29.46% to 211.35% for fruit weight, respectively. GCV contributed significantly towards PCV over the environmental coefficient of variation for all the traits. All the traits have shown high broad-sense heritability (> 90%). The percent GA ranged from 1.82% to 279.79%. However, GA as a percentage of the mean (GAM) ranged from 56.65% (leaf length) to 434.08% (fruit weight). Among the traits, the maximum GAM was observed for yield, and attributing traits are fruit weight, fruit diameter, the number of seeds per fruit, fruit length, and yield per plant.

**Table 2:** Estimation of genetic parameters for different traits in *Capsicum* spp.

Traits	Mean ± SE	Range		GCV (%)	PCV (%)	h² (Broad Sense)	Genetic Advance (%)	GAM
		Minimum	Maximum					
Fruit weight (g)	4.73 ± 0.38	0.27	69.00	211.03	211.35	99.0	20.56	434.08
Fruit length (cm)	4.84 ± 0.33	0.60	13.55	55.04	55.90	97.0	5.41	111.64
Fruit dia. (cm)	1.26 ± 0.09	0.35	5.15	70.35	71.10	98.0	1.82	143.39
Leaf length (cm)	9.91 ± 0.53	5.56	17.40	28.46	29.46	93.0	5.62	56.65
Leaf width (cm)	4.69 ± 0.25	2.10	11.10	47.86	48.48	97.0	4.57	97.32
Leaf area (cm^2^)	67.96 ± 2.52	22.55	170.05	50.89	51.17	99.0	70.88	104.28
No of fruits/plant	73.37 ± 5.24	9.00	217.50	48.28	49.34	96.0	71.42	97.33
Yield per plant (g)	233.50 ± 12.73	18.48	748.00	58.67	59.18	98.0	279.79	119.82
No of seeds/fruit	43.01 ± 2.05	4.50	142.50	61.67	62.04	99.0	54.32	126.29

GCV, genotypic coefficient of variation; PCV, phenotypic coefficient of variations; h^2^, heritability; and GAM, genetic advance as percentage of mean.

### 3.4 Genetic diversity based on molecular markers

#### 3.4.1 Allelic diversity

Molecular analysis using 47 SSR markers (**Table 3**) showed wider allelic variations among the genotypes of *Capsicum* spp. (**Supplementary Figure 1**). All the markers were found to be polymorphic, and the average number of alleles per locus ranged from 2 (HPMSE-7) to 10 (HPMSE-5) (mean = 4.89) in the group of 106 individuals having five populations. The PIC was also high (0.61) and ranged from 0.20 (HPMSE-7) to 0.847 (CAMS-91). The values of the Shannon information index were higher than 1.5, and their corresponding PIC value was also above 0.7. The highest PIC and Shannon information index was observed for the markers CAMS-091 (0.84 and 2.027), HPMSE-72 (0.79 and 1.80), and HPMSE-5 (078 and 1.83). The observed heterozygosity was lower than the expected heterozygosity and ranged from 0.00 to 1.0. Similarly, the minor allele frequency ranged from 0.179 (CAMS-91) to 0.870 (HPMSE-7). All the SSRs showed a significant Chi square test for the HWE test at *P* < 0.001.

**Table 3:** Details of microsatellite markers used in *Capsicum* spp.

Marker	Na	Ne	A	PIC	I	Ho	He	F	MAF	HWE*χ* ^2^	*p* value
HPMSE2	4	2.40	0.981	0.541	1.102	0.029	0.583	0.951	0.601	284.21	0.000***
HPMSE5	10	5.30	0.981	0.786	1.831	0.202	0.811	0.751	0.298	39.58	0.000***
HPMSE7	2	1.29	0.981	0.200	0.386	0.087	0.226	0.617	0.870	630.02	0.000***
HPMSE8	3	1.65	1.000	0.323	0.618	0.000	0.394	1.000	0.736	212.00	0.000***
HPMSE14	4	2.44	1.000	0.526	1.011	0.057	0.590	0.904	0.561	182.80	0.000***
HPMSE16	5	3.15	1.000	0.639	1.347	0.275	0.682	0.598	0.480	468.58	0.000***
HPMSE17	6	3.97	1.000	0.716	1.569	0.047	0.748	0.937	0.415	424.00	0.000***
HPMSE21	3	2.91	1.000	0.582	1.083	0.000	0.656	1.000	0.415	530.00	0.000***
HPMSE23	5	2.48	0.953	0.554	1.170	0.000	0.598	1.000	0.585	145.29	0.000***
HPMSE26	5	2.82	1.000	0.580	1.190	0.000	0.645	1.000	0.462	537.04	0.000***
HPMSE31	3	2.74	1.000	0.562	1.052	0.000	0.635	1.000	0.472	525.96	0.000***
HPMSE40	6	3.88	0.991	0.708	1.545	0.000	0.742	1.000	0.415	474.52	0.000***
HPMSE54	6	4.75	1.000	0.756	1.627	0.019	0.789	0.976	0.271	408.91	0.000***
HPMSE56	6	2.39	1.000	0.548	1.207	0.075	0.581	0.870	0.613	212.00	0.000***
HPMSE58	3	2.44	0.915	0.508	0.969	0.000	0.590	1.000	0.509	149.58	0.000***
HPMSE62	5	4.08	0.962	0.715	1.498	0.289	0.755	0.618	0.325	177.02	0.000***
HPMSE66	4	1.98	0.991	0.415	0.829	0.085	0.496	0.829	0.637	315.00	0.000***
HPMSE67	4	2.71	1.000	0.575	1.144	0.000	0.631	1.000	0.524	212.00	0.000***
HPMSE69	6	4.76	0.981	0.758	1.656	0.406	0.790	0.486	0.277	171.31	0.000***
HPMSE70	3	2.48	1.000	0.529	0.999	0.058	0.596	0.903	0.548	420.14	0.000***
HPMSE71	6	3.85	1.000	0.699	1.498	0.075	0.740	0.898	0.368	212.47	0.000***
HPMSE72	8	5.48	1.000	0.792	1.801	0.151	0.817	0.815	0.241	424.00	0.000***
HPMSE73	3	1.89	1.000	0.397	0.773	0.000	0.472	1.000	0.667	363.32	0.000***
HPMSE74	7	4.37	1.000	0.738	1.632	0.066	0.771	0.914	0.344	424.00	0.000***
HPMSE94	5	3.20	1.000	0.627	1.266	0.000	0.687	1.000	0.368	212.00	0.000***
HPMSE101	5	3.43	1.000	0.658	1.367	0.057	0.708	0.920	0.377	424.00	0.000***
HPMSE102	5	3.27	0.991	0.650	1.359	0.000	0.694	1.000	0.462	210.00	0.000***
CAeMS009	5	2.20	0.981	0.486	0.983	0.000	0.546	1.000	0.613	416.00	0.000***
CAeMS015	4	3.25	0.991	0.636	1.264	0.000	0.693	1.000	0.368	420.00	0.000***
CAMS032	5	3.29	1.000	0.642	1.321	0.353	0.696	0.493	0.382	592.01	0.000***
CAeMS035	4	2.21	1.000	0.478	0.947	0.035	0.547	0.936	0.599	318.00	0.000***
CAeMS060	4	3.75	0.962	0.684	1.351	0.000	0.733	1.000	0.330	90.66	0.000***
CAMS066	6	2.84	1.000	0.603	1.276	0.676	0.648	-0.044	0.524	318.00	0.000***
CAeMS068	6	4.51	0.972	0.746	1.633	0.010	0.778	0.988	0.340	497.50	0.000***
CAMS071	5	3.49	0.991	0.664	1.381	0.009	0.714	0.987	0.368	70.78	0.000***
CAeMS073	3	2.45	1.000	0.526	0.993	0.000	0.591	1.000	0.557	212.00	0.000***
CAMS091	8	7.29	1.000	0.847	2.027	1.000	0.863	-0.159	0.179	220.69	0.000***
CAeMS138	5	3.17	0.962	0.636	1.327	0.000	0.685	1.000	0.457	714.00	0.000***
CAeMS144	5	3.00	0.962	0.598	1.166	0.000	0.667	1.000	0.394	119.76	0.000***
CAMS179	7	4.44	1.000	0.742	1.663	0.038	0.775	0.951	0.340	318.00	0.000***
CAMS212	4	3.37	0.981	0.644	1.267	0.000	0.703	1.000	0.340	208.00	0.000***
CAMS368	5	2.95	1.000	0.597	1.190	0.278	0.661	0.580	0.450	400.18	0.000***
CAMS396	8	3.83	0.849	0.697	1.549	0.000	0.739	1.000	0.343	144.86	0.000***
CAMS406	3	2.14	0.991	0.476	0.913	0.000	0.533	1.000	0.632	315.00	0.000***
CAMS452	3	2.93	1.000	0.585	1.087	0.000	0.658	1.000	0.404	424.00	0.000***
CAMS493	4	3.14	1.000	0.615	1.192	0.000	0.681	1.000	0.352	212.00	0.000***
CAMS823	4	3.93	0.811	0.698	1.377	0.529	0.745	0.290	0.289	160.68	0.000***
Mean	4.89	3.28	–	0.610	1.265	0.104	0.661	0.851	0.449	–	

NA, number of alleles; Ne, effective number of alleles; A, availability; PIC, polymorphism information content; I, Shannon’s information index; Ho, observed heterozygosity; He, expected heterozygosity; F, fixation index; MAF, major allele frequency; HWE, deviation from Hardy–Weinberg equilibrium with significant at ***p < 0.001.

#### 3.4.2 Population diversity

**Figure 4:**
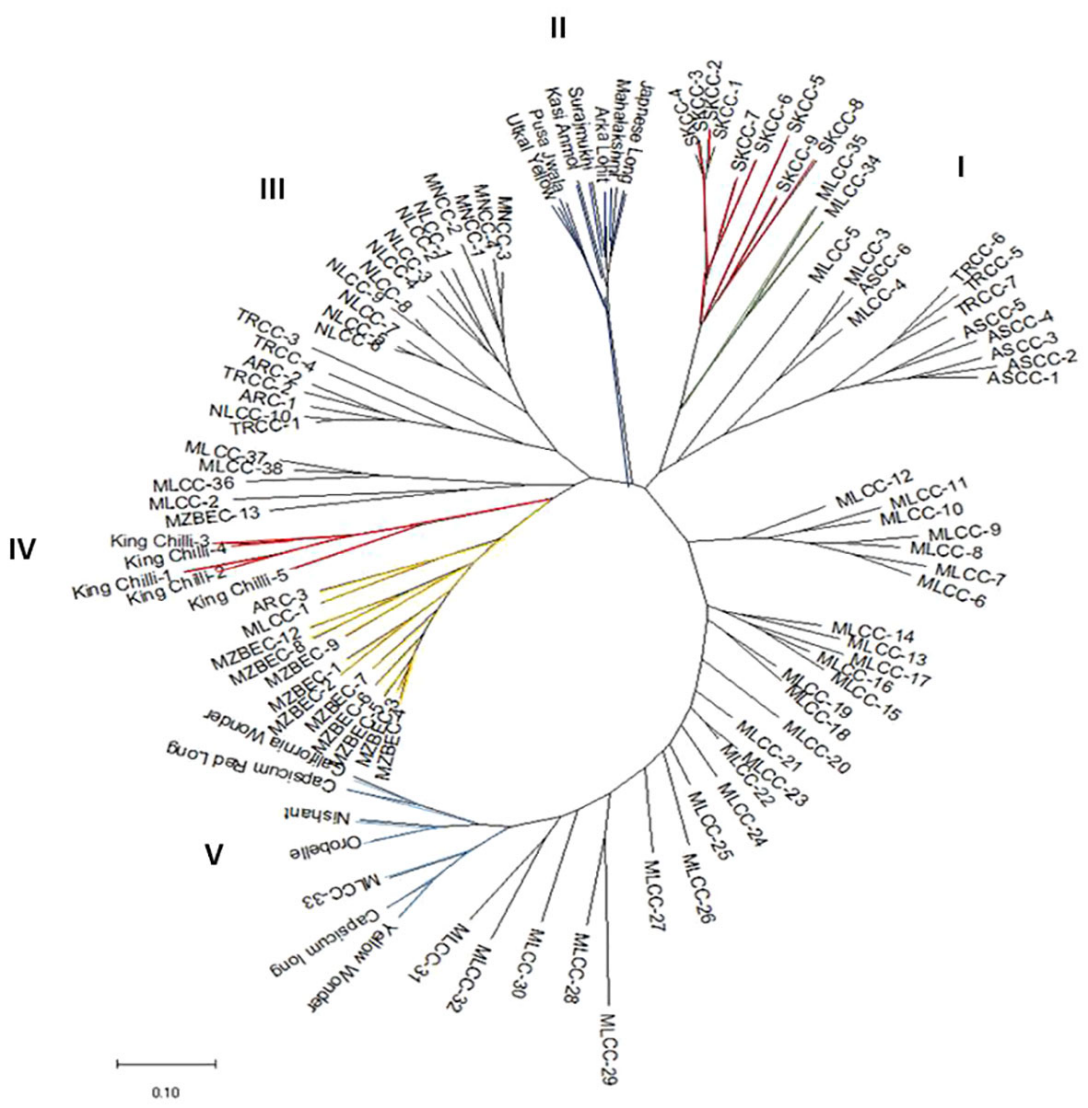
Neighbor-joining dendogram showing genetic relationships among the accessions of *Capsicum* spp. based on Nei’s genetic distance using 47 SSRs markers. Line colors differentiate the genotypes: *Capsicum annuum* (cherry chilli; green line), *C. annuum* (dalle-chillies; dark red line), commercial cultivar of *C. annuum* (hot pepper; dark blue lines), *C. chinense* (king-chillies; red lines), *C. frutescens* (bird’s eye chillies; yellow lines), *C. annuum* (sweet pepper; blue lines), and other landraces of *C. annuum* (hot pepper; black lines).

The analysis of molecular variance (**Table 4**) showed the existence of significant variation within (69%) and between (31%) the populations of *Capsicum* spp. The genetic differentiation (*Gst*) among the population was 0.394, which indicates that 60.60% genetic variation is within populations and only 39.4% between populations. Among the populations, wider heterozygosity was also observed, and it ranged from 0.00 to 1.0. The maximum genetic distance (1.303) was observed between dalle-chilli (*C. annuum*) and king-chilli (*C. chinense*), followed by king-chilli and sweet pepper (1.276), as well as dalle-chilli and sweet pepper (1.227). However, the lowest genetic distance was between sweet pepper and hot pepper (**Table 5**). The cluster analysis has also revealed wider diversity in different *Capsicum* landraces in the region (**Figure 4**). All 106 genotypes were grouped into five major clusters, and commercial cultivars of hot pepper were found to be closer to each other and grouped together. Similarly, the genotypes of sweet pepper were also grouped together, and the genotypes of bird’s eye chilli and king-chilli were found closer to each other.

**Table 4:** Analysis of molecular variations (AMOVA).

Source of variations	df	SS	MS	EV	%
Among populations	5	1,449.456	289.891	21.446	31%
Within populations	100	4,734.375	47.344	47.344	69%
Total	105	6,183.830		68.790	100%

df, degree of freedom; SS, sum of squares; MS, mean squares; EV, estimated variance.

**Table 5:** Pairwise Nei genetic distance among the landraces of *Capsicum* spp.

	Dalle-chilli (*C. annuum*)	King-chilli (*C. chinense*)	Bird’s eye chili (*C. frutescens*)	Cherry chilli (*C. annuum*)	Hot pepper (*C. annuum*)
King-chilli (*C. chinense*)	1.303				
Bird’s eye chili (*C. frutescens*)	0.766	0.598			
Cherry chilli (*C. annuum*)	0.652	1.218	1.089		
Hot pepper (*C. annuum*)	0.696	0.717	0.517	0.764	
Sweet pepper (*C. annuum*)	1.227	1.276	0.999	1.094	0.423

#### 3.4.3 Principal coordinate analysis

**Figure 5:**
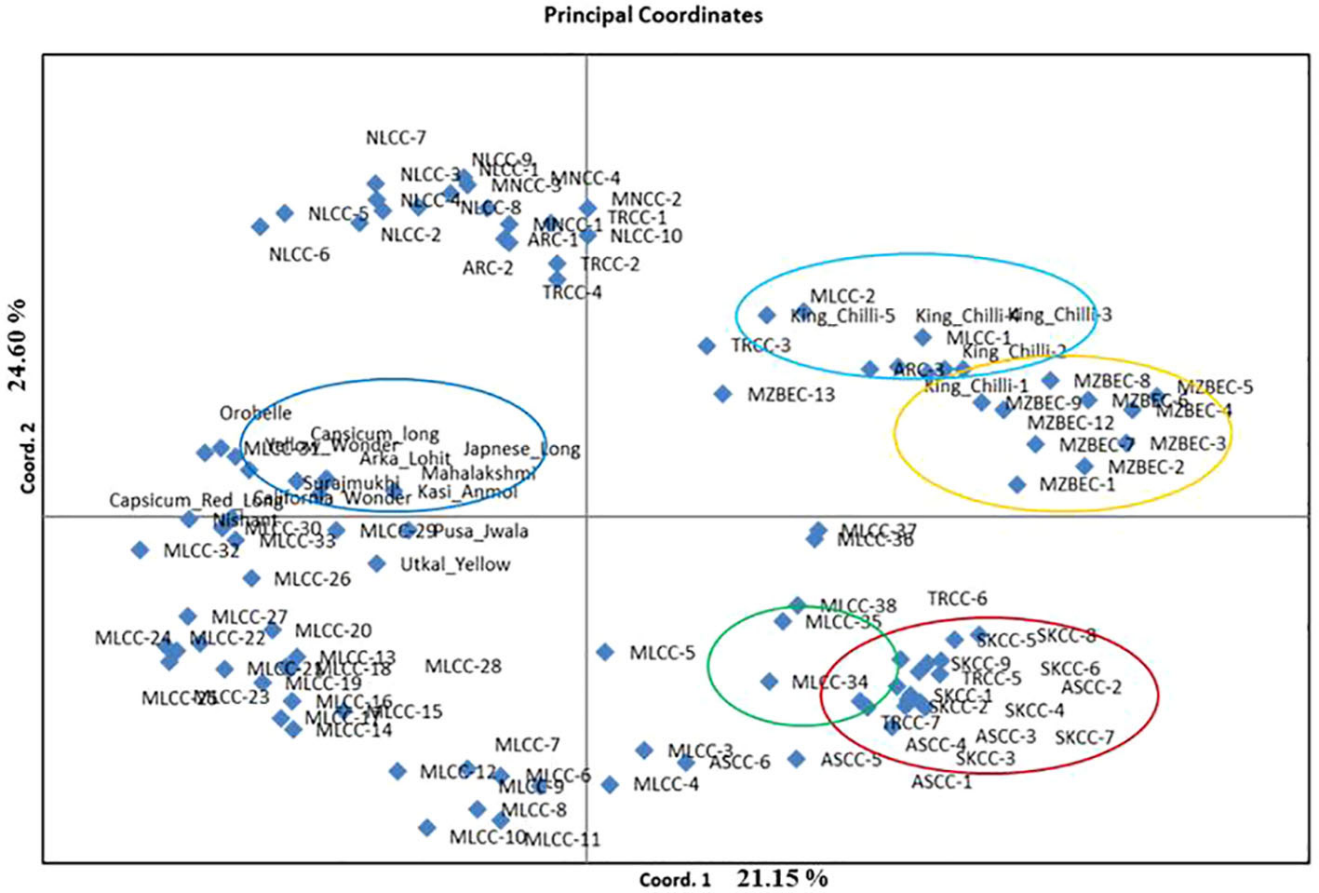
Principal coordinate analysis (PCoA) plot of accessions of *Capsicum* spp. Circle colors indicate the grouping of the genotypes: *Capsicum annuum* (cherry chilli; green line), *C. annuum* (dalle-chillies; dark red line), commercial cultivar of *C. annuum* (hot pepper and sweet pepper; dark blue lines), *C. chinense* (king-chillies; light blue), and *C. frutescens* (bird’s eye chillies; yellow lines).

The PCoA revealed that the first three PCoA explained 65.22% of the total variation, with 25.15% defined by the first coordinate and 24.60% by the second coordinate. The first coordinate separated all the local landraces of dalle-chilli (*C. annuum*, red circle), bird’s eye chilli (*C. frutescens*, yellow circle), and king-chilli (*C. chinense*, light blue circle) to hot pepper and sweet pepper (**Figure 5**). Similarly, the second coordinate separated bird’s eye chilli and king-chilli to *C. annuum* (dalle-chilli and cherry chilli) as indicated by green circle in **Figure 5**. Moreover, the local landraces of the hot pepper (*C. annuum*) were found most diverse and distributed across the coordinates.

#### 3.4.4 Multiple correspondence analyses

**Figure 6:**
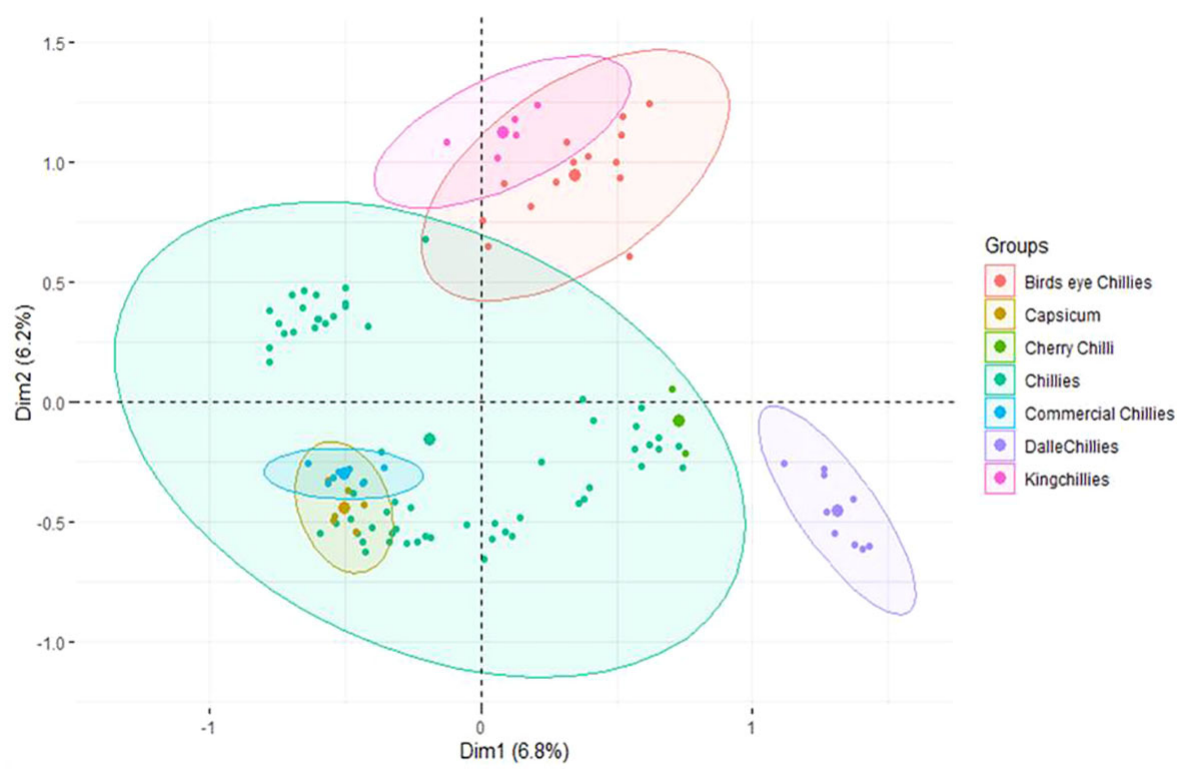
Multiple correspondence analysis of the 106 genotypes of *Capsicum* spp. based on groups of the genotypes. The species of the groups are *C. frutescens* (bird’s eye chillies), *C. annuum* (Capsicum, cherry chilli, chillies, and commercial chillies), *C. annuum* (dalle-chillies), and *C. chinense* (king-chillies).

The first three principle components (PCs) identified by MCA have explained only 6.8%, 6.20%, and 5.42% of the population variance, respectively (**Figure 6**). Like PCoA, the PC-I of MCA also differentiated the landrace dalle-chille (*Capsicum annuum*) from the other groups, whereas the PC-II has differentiated the landrace king-chilli (*C. chinense*) and bird’s eye chilli (*C. frutescens*) from the other group of *C. annuum* (Sweet pepper, cherry chilli, and commercial cultivars of the hot pepper). The landraces of hot pepper (*C.annuum*) were distributed across the PCs. Moreover, the landraces of bird’s eye-chilli and king-chilli were found close to each other.

#### 3.4.5 Genetic structure and interrelationship

**Figure 7:**
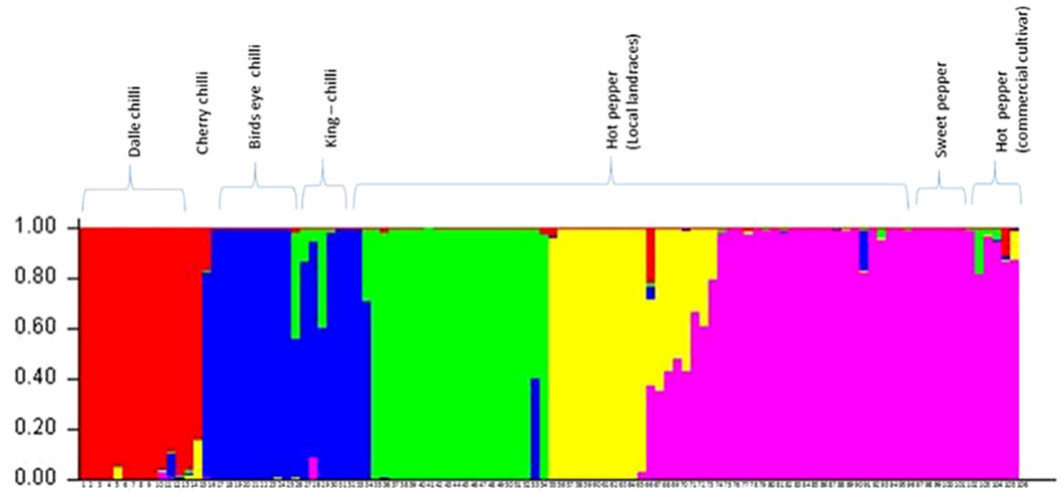
Population structure (at Δ K = 5) of the accessions of *Capsicum* spp. based on SSR markers. The species of the genotypes are *C. annuum* (dalle-chillies), *C. annuum* (cherry chilli), *C. frutescens* (bird’s eye chillies), *C. chinense* (king-chillies), and *C. annuum* (hot pepper and sweet pepper).

The optimum cluster number was determined as per the procedure described by Evanno et al. (2005) using software STRUCTURE. The analysis detected the maximal ΔK at K=5 (**Supplementary Figure 2**). Clusters differentiated perfectly between and within the *Capsicum* species (**Figure 7**). The proportions of genotypes with admixture were few, and it was more common in hot pepper.

### 3.5 Comparative performance of *Capsicum* landraces for quality traits

**Figure 8:**
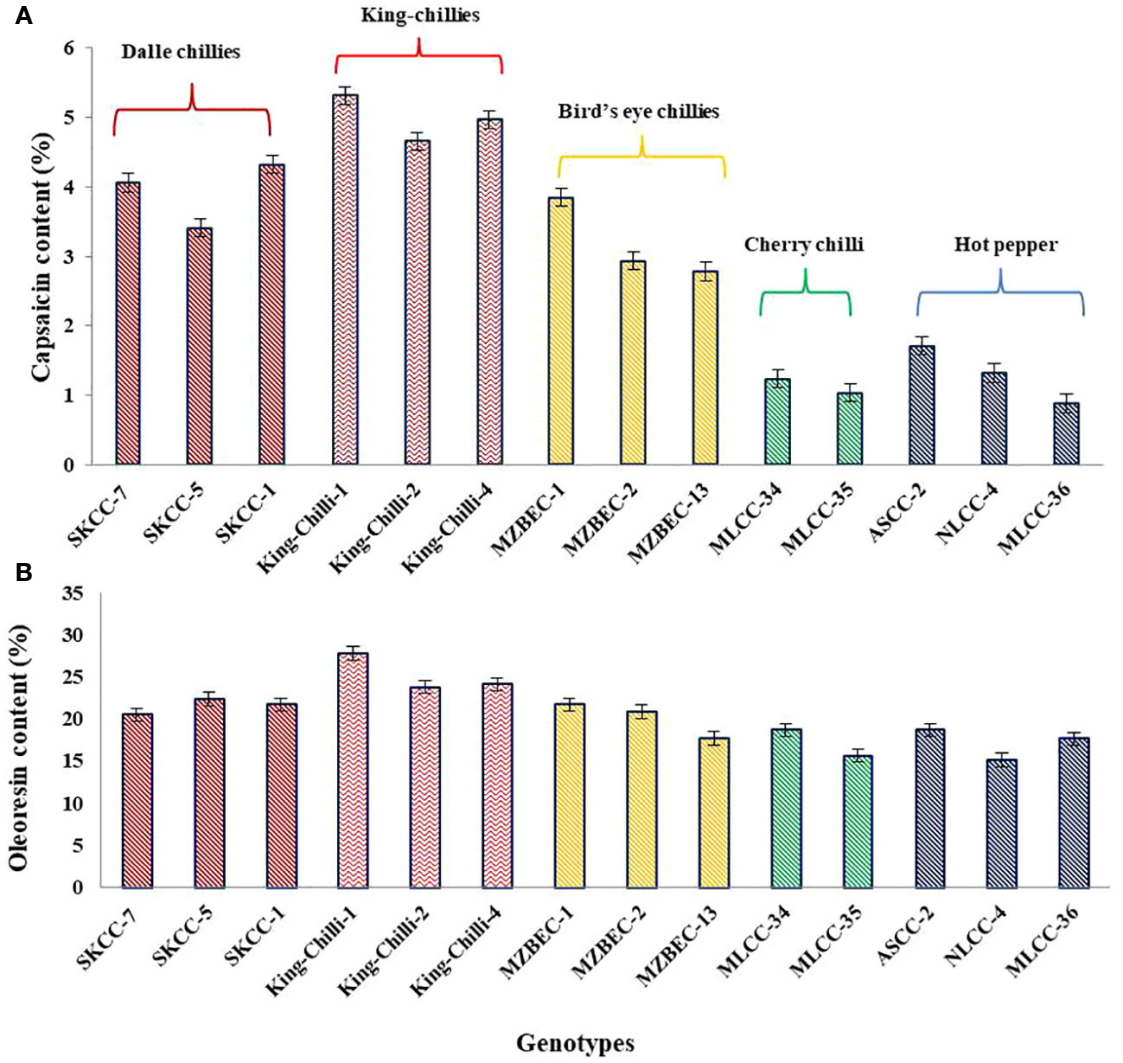
Comparative performance of high yielding *Capsicum* landraces for quality traits. **(A)** Capsaicin content and **(B)** oleoresin content. The species of the groups are *C. annuum* (dalle-chillies), *C*. *chinense* (king-chillies), *C*. *frutescens* (bird’s eye chillies), *C*. *annuum* (cherry chillies), and *C*. *annuum* (hot pepper).

The results presented in **Figure 8** have shown the presence of significant variations within and between the groups of the *Capsicum* landraces for both capsaicin and oleoresin contents. Among the landraces, the maximum average capsaicin content was observed in king-chillies (*C. chinense*; 4.98%), followed by dalle-chillies (*C. annuum*; 3.93%) and bird’s eye chillies (*C. frutescens*; 3.18%). However, the minimum capsaicin content was recorded in the cherry chillies (*C. annuum*; 1.13%). Similarly, the oleoresin content was also higher in king-chillies (*C.chinense*; 25.24%), followed by dalle-chillies (*C. annuum*; 21.56%), bird’s eye chillies (*C. frutescens*; 21.13%), cherry-chillies (*C. annuum*; 17.21%), and hot pepper (*C. annuum*; 16.96%).

## 4 Discussion

The NE region of India is considered one of the biodiversity hotspots globally (Thamburaj and Singh, 2003; Adluri et al., 2017; Colney et al., 2018; Vaishnavi et al., 2018; Baruah et al., 2019; Mena et al., 2019) and has diverse climatic ranges, from tropical to alpine (Verma et al., 2023). *Capsicum* spp. has wider adoptability and is grown throughout the country. The GI tag landraces of the region are known for their distinct agro-morphological characteristics and unique quality traits, performing well in a particular niche area. This is due to the favorable agro-climatic conditions of the region, which comes under a humid subtropical climate with relative humidity (81.2%–91.5% in the morning and 52.6%–81.8% in the evening) during the crop period. The results of the CCA have also shown the significant relationship between the climatic variables (temperature) of the niche area with landraces and the quantitative traits. The landraces of cool to mild-warm climate were differentiated from the landraces adapted to warm climatic conditions. The ecology of the niche area of dalle-chillies (*C. annuum*) is characterized by a cool and humid climate during the crop period (March to October). Compared to other landraces, dalle-chilli is adapted to low temperatures and goes dormant during the winter, when the average minimum temperature is 2.9°C–6.3°C in their niche area. Likewise, the niche area (Nagaland, Assam, and Manipur) of landrace king-chilli has shown mildly warmer than the weather parameters of a niche area of dalle-chilli. We could also observe poor fruit setting (< 43.3% ± 4.2%) under weather conditions (mint < 8.0°C; maxT < 23.5°C; minRH = 48.8%; maxRH = 72.3%) at our experimental farm during the winter. Under these conditions, the fruits even after fruit sets failed to develop; remaining small, red-colored fruits without seeds and this could be due to a lack of pollination and fertilization. Similarly, the climate of the niche area of bird’s eye chilli (*C. frutescens*) is characterized by a cool to warm and humid climate (Mizoram, Manipur, and Meghalaya), during the cropping period. The range of weather parameters (temp) has shown comparatively, the wider adoptability of the bird’s eye chillies over the king-chillies (*C. chinense*) and dalle-chillies (*C. annuum*). Due to its wider adaptability, it is also grown in other parts of the country, such as Kerala, Karnataka, Maharashtra, and Andhra Pradesh under the warm and humid climatic conditions (Thamburaj and Singh, 2003). The yield and quality of *Capsicum* spp. are highly influenced by genotype and environment (Tiwari et al., 2005; Gurung et al., 2012); hence, these landrace can be grown year-round under protected conditions by maintaining the growing environments of their niche area during the cropping period, and there is also a possibility for area expansion to other parts of the world having similar agro-climatic conditions.

A wider variability was observed for fruit color, shape, and size of leaves and fruits, as well as other yield attributes, especially in the niche area of the landraces. This may be due to adaptability to the varied climate of the region, natural crossings, and selection. Similarly, the broader variability in niche area was also reported earlier in bird’s eye chilli (*C. frutescens*) (Dutta et al., 2017; Vaishnavi et al., 2018; Santhosha et al., 2019) and king-chilli (*C. chinense*) (Bhagowati and Changkija, 2009). The GCV is an accurate indicator of the extent of genetic variability in the population. Our study exhibited that all the quantitative traits had a slightly higher PCV than the GCV, indicating the influence of some degree of the environment on the phenotypic expression of these characters. It suggests that selection based on all these traits would be helpful for future crossing programs. Furthermore, all the traits showed high heritability coupled with high GA, except for leaf length, which indicates the strong influence of an additive gene on these traits is under action. Hence, simple selection based on the phenotypic performance of these traits would be more effective and efficient. The findings are in congruence with those reported earlier in *Capsicum* spp (Manju and Sreelathakumary, 2002; Pandiyaraj et al., 2017; Solomon et al., 2019). Fruit size is an important economic trait; for export, large fruit sizes usually fetch premium prices, especially in king-chilli (*C. chinense*), bird’s eye chilli (*C. frutescens*), and dalle-chilli (*C. annuum*). Hence, genotypes SKCC-5 and SKCC-4 of dalle-chilli, King-chilli-4 and King-chilli-1 of king-chilli, and MZBEC-13 and MLCC-2 of bird’s eye chilli having large fruit sizes could be promoted for the export market. Furthermore, fruit length, diameter, weight, number of fruits per plant, and yield in *Capsicum* spp. are governed by additive gene action and responsive to selection (Manju and Sreelathakumary, 2002; Rosmaina et al., 2016; Singh et al., 2017), and the superior genotypes for these traits can be used for future hybridization and selection. Moreover, identified high-yielding genotypes of the dalle-chilli (SKCC-7, SKCC-5, and SKCC-1), king-chilli (King-chilli-1 and King-chilli-4), cherry chilli (MLCC-34), bird’s eye chilli (MZBEC-13, MZBEC-1, and MLCC-1), hot pepper (MLCC-36, ASCC-2, NLCC-4, and NLCC-10), and sweet pepper (California Wonder) can be promoted for commercial production.

Our results of molecular analyses have shown the presence of wider genetic diversity among the accessions of *Capsicum* spp. This high diversity has also been proven by the higher (4.89) average number of alleles per locus as compared to 2.78 and 3.5 alleles per locus reported by Dhaliwal et al. (2014) and Hanácek et al. (2009), respectively. PIC estimates the discriminating power of a marker by taking into account the number of alleles at a locus and the relative frequencies of these alleles. The PIC value depends on the genetic diversity among the populations. The present study showed the PIC value varied (0.20–0.85) with an average of 0.61, which was greater than the value reported by many researchers (Minamiyama et al., 2006; Yumnam et al., 2012; Sharmin et al., 2018) but lower than the value of 0.76 described by Lee et al. (2016) from diverse *Caspsicum* spp. According to Xie et al. (2010), high, medium, and low locus polymorphisms are defined as PIC > 0.5, PIC 0.25–0.5, and PIC < 0.25, respectively. Accordingly, PIC in our investigation indicated a wider genetic base due to high locus polymorphism. The results of a higher PIC value coupled with a higher Shannon information index also prove the locus diversity in the population.

*Capsicum* spp. is considered an often cross-pollinated crop; 25 SSR loci have shown heterozygosity among the genotypes. The extent of genetic variations is also measured as the amount of actual or potential heterozygosity existing in the population. The observed heterozygosity was lower than the expected value, showing a departure from HWE and the possibility of inbreeding for a long time under different geographical conditions due to natural barriers and isolation, which could be attributed to the interplay of different factors such as artificial selection, selective collection, non-random mating between individuals, population structure and size, and the Wahlund effect, i.e., mixing of individuals from different genetic sources (Johnson and Black, 1984; Hernández-Verdugo et al., 2001). Similarly, Lee et al. (2016) also observed lower values for observed heterozygosity in four domesticated species (*C*. *annuum*, *C*. *baccatum*, *C. chinense*, and *C. frutescens*) and two wild species (*C. chacoense* and *C. eximium*), whereas *C. cardenasii*, *C. galapagoense*, *C. pratermissum*, *C. pubescens*, and *C. tovarii* had relatively higher values.

From cluster analysis, the landraces bird’s eye chilli (*C. frutescens*) and king-chilli were found to be closer to each other in our study. Pereira-Dias et al. (2019) also reported kinship between *C. chinense* and *C. frutescens.* However, landraces of dalle-chilli (*C. annuum*) were found closer to hot pepper and sweet pepper (*C. annuum*). Colney et al. (2018) also reported a close association between *C. annuum* and dalle-chilli, whereas Cheng et al. (2016) found *Capsicum annuum* closer to *C. frutescens* than *C. chinense.* Furthermore, cherry-chilli (*C. annuum*) genotypes (MLCC-34 and MLCC-35) clustered together with landraces of dalle-chilli (SKCC-1 to SKCC-8) and were found closer to *C. annuum*. This indicates that the landrace dalle-chilli, an allotetraploid (Jha et al., 2017), evolved as a naturally fertile *C. annuum*. In contrast, Indira and Abraham (1977) reported sterility in tetraploid *C. annuum* developed through induced mutation. Among the genotypes of commercial cultivars of both *C. annuum* (hot and sweet pepper), they were found to be closer to each other in their respective groups. Among the populations based on pair-wise Nei genetic distance, the maximum genetic distance (1.30) was observed between king-chilli (*C. chinense*) and bird’s eye chilli (*C. frutescens*), followed by king-chilli and sweet pepper (1.27) and dalle-chilli and sweet pepper. The maximum genetic distance between the landraces may be due to associated with different geographical origins, poor cross-compatibility (Martins et al., 2015), and selection and polyploidy in dalle-chilli. While the least genetic distance was between hot pepper and sweet pepper, this could be due to free gene flow among the *C. annuum* (hot pepper and sweet pepper) genotypes.

The results of both AMOVA and G*st* analyses have confirmed the presence of wider diversity within and between the populations of *Capsicum* spp. The results of model-based clustering using STRUCTURE 2.3.4 agreed with Nei’s genetic distance-based clustering, PCoA, and multiple correspondence analyses. Among the genotypes, the proportions of genotypes with admixture were few, possibly due to the different geographical origins. The admixture in landraces of hot pepper (*C. annuum*) and king-chilli (*C. chinense*) may be due to free natural gene flow between them, hybridization, and selection. Due to open pollination and selection in different ecogeographic regions, variability is maintained. Gu et al. (2019) also observed the genetic differentiation in the *Capsicum* genotypes for fruit type and geographical distribution. Landrace king-chilli is considered as an interspecific hybrid between *C. chinense* and *C. frutescens* based on molecular analysis (Bosland and Baral, 2007; Verma et al., 2018a; Malakar et al., 2019). In our study, the structure analysis has shown admixture between king-chilli (*C. chinense*) and bird’s eye chilli (*C. frutescens*). This could be due to the closeness and cross-compatibility between *C. chinense* and *C. frutescens* (Nicolaï et al., 2013). Moreover, the presence of calyx teeth in king-chilli morphologically differentiates it from *C. chinense*. Furthermore, landrace dalle-chilli (*C. annuum*) constituted a separate group with the least admixture and was found to be closer to *C. annuum*. However, based on flower characters, i.e., light yellow-green corolla and teeth on the calyx, indicate closeness to *C. frutescens* and differentiation from *C. chinense*. Jha et al. (2017) reported dalle-chilli as an allopolyploid with an absolute difference from the diploid members of *C. annuum*. The genetic similarity with *C. annuum* and morphological similarity with *C. frutescens* indicate that dalle-chilli probably evolved naturally as an interspecific hybrid between *C. annuum* and *C. frutescens.*

Comparatively, among the selected landraces, king-chilli (*C. chinense*) and dalle-chilli (*C. annuum*) were found to be superior for economically important traits, capsaicin, and oleoresin content, over the bird’s eye chilli (*C. frutescens*) and hot pepper (*C. annuum*). Similar findings were also observed by Mini (Pandey et al., 2008) and Jyoti et al (Mini, 1997). in different *Capsicum* species. These superior landraces can be promoted for commercial production in their niche areas for value-added products like oleoresin and capsaicin extraction and further export to international markets.

## 5 Conclusion

Our study showed that certain pockets of the NE region are a center of the evolution and hot spot of unique GI tag landraces (king-chilli, *C.chinense*; bird’s eye chilli, *C. frutescens*; and dalle-chilli, *C. annuum*) of the *Capsicum* species. The evolution of these landraces occurs through natural interspecific hybridization and selection and is adapted to the different ecology of the region, characterized as cool-humid and moderately warm-humid climates for dalle-chilli (*C. annuum*) and king-chilli (*C. chinense*), respectively. The climatic variables (temperature) have shown significant impact on agro-morphological traits and adoptability of the landraces to their niche areas of the region. Both quantitative traits and molecular analysis have shown wider variability for traits and diversity within and between the groups. High heritability and GA for all the fruit traits have also indicated that these traits are highly responsive to selection. The molecular analyses and flowering features of landrace king-chilli (*C.chinense*) also suggested that it was more closely related to bird’s eye chilli (*C. frutescens*), confirming their evolution in the niche area (Nagaland, Manipur, and parts of Upper Assam) as an interspecific hybrid between *C. chinense* and *C. frutescens*. Dalle-chilli (*C. annuum*) was reported as an allotetraploid species and was found to be genetically closely related to *C. annuum*. Considering the flower traits, dalle-chilli probably originated from the natural cross between *C. annuum* and *C. frutescens* in the niche area of Sikkim and West Bengal. Dalle-chilli, a winter-hardy landrace that is also grown as a perennial crop in backyards, can be utilized in varietal development programs against abiotic stresses like low temperatures and frost. Furthermore, the identified superior genotypes among the landraces can be promoted for *in situ* conservation, production, value addition, and sources of desirable genes in niche areas as well as in suitable climatic conditions in other parts of the country and world.

## Data availability statement

The original contributions presented in the study are included in the article/**Supplementary Material**. Further inquiries can be directed to the corresponding author.

## Author contributions

VV: Writing – original draft, Supervision, Software, Resources, Methodology, Investigation, Formal analysis, Data curation, Conceptualization. AP: Writing – review & editing, Visualization, Software, Investigation, Formal analysis, Data curation. AT: Writing – review & editing, Resources. HR: Writing – review & editing. ND: Writing – review & editing, Data curation. AK: Writing – review & editing, Formal analysis. TB: Writing – review & editing, Resources. AJ: Writing – review & editing, Supervision, Investigation. VM: Writing – review & editing, Resources.
