# Supplementary material for: Ecology, genetic diversity, and population structure among commercial varieties and local landraces of Capsicum spp. grown in northeastern states of India
Source: Front Plant Sci. 2024 Apr 4;15:1379637. doi: 10.3389/fpls.2024.1379637 (PMC11024323; doi:10.3389/fpls.2024.1379637)
Supplement: Supplementary file 1 [file DataSheet_1.docx]

*
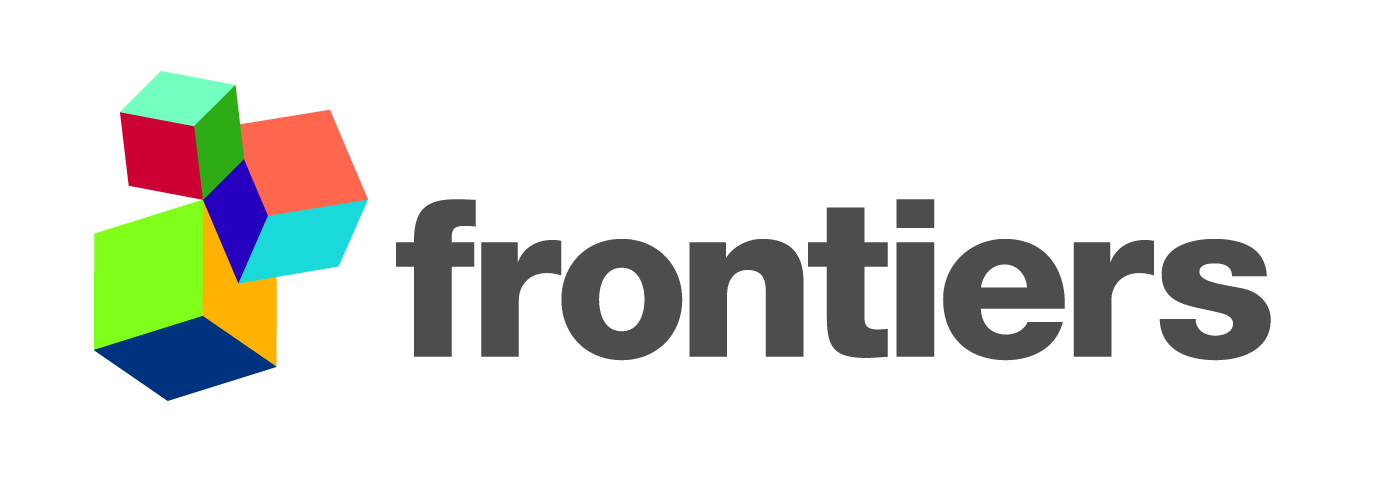
****Supplementary Material***

**Ecology, genetic diversity and population structure among commercial varieties and local landraces of Capsicum spp. grown in northeastern states of India**

Veerendra Kumar Verma^1^*, Avinash Pandey^2^, A. Thirugnanavel^3^, Heiplanmi Rymbai^1^, Niharika Dutta^1^, Amit Kumar^1^, Tshering Lhamu Bhutia^4^, Anjani Kumar Jha^5^ and Vinay Kumar Mishra^1^

^1^ICAR Research Complex for North Eastern Hill Region, Umroi Road, Umiam-793103, Meghalaya, India.

^2^School of Genomics and Molecular Breeding, ICAR-Indian Institute of Agricultural Biotechnology (IIAB), Ranchi-834003, Jharkhand, India.

^3^ICAR - Central Citrus Research Institute, Post Box No. 464, Amravati Road, Nagpur - 440033, Maharashtra, India.

^4^Scientist Horticulture, ^1^ICAR Research Complex for NEH Region, Sikkim Centre, Tadong, Gangtok-737 102, Sikkim, India

^5^Principal Scientist-Horticulture, ICAR-Indian Institute of Horticultural Research, Hessaraghatta Lake Post, Bengaluru-560089, India.

***Corresponding author postal address:**

Dr. Veerendra Kumar Verma

Senior Scientist- Horticulture

ICAR Research Complex for NEH Region, Umroi Road, Umiam 793103, Meghalaya, India.

Email: [verma.veerendra@gmail.com](mailto:verma.veerendra@gmail.com)

# Supplementary Figures and Tables

## Supplementary Figures

**
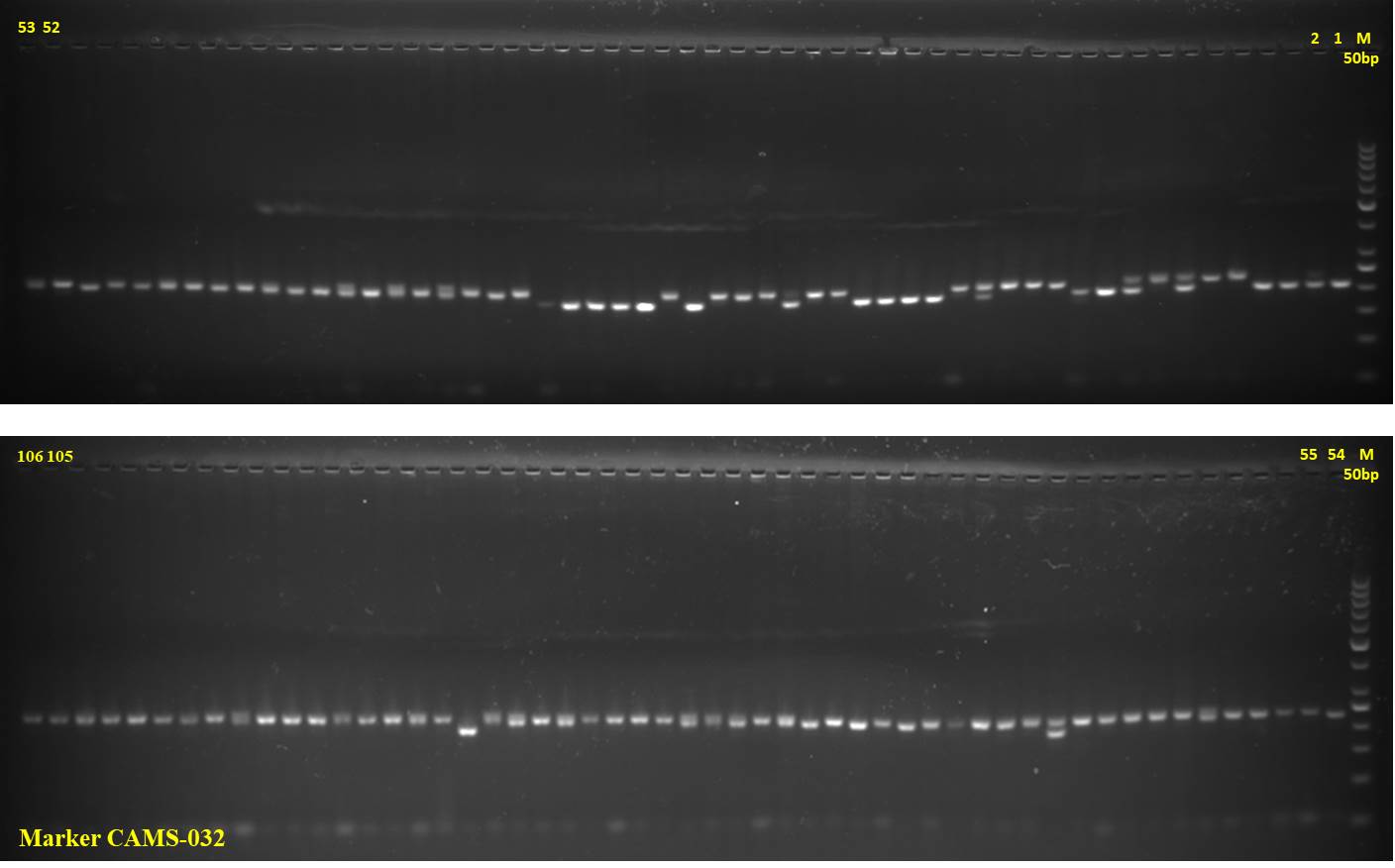
**

Supplementary Figure 1. Allelic variations among the genotypes of *Capsicum* spp. based on microsatellite marker CAMS-032


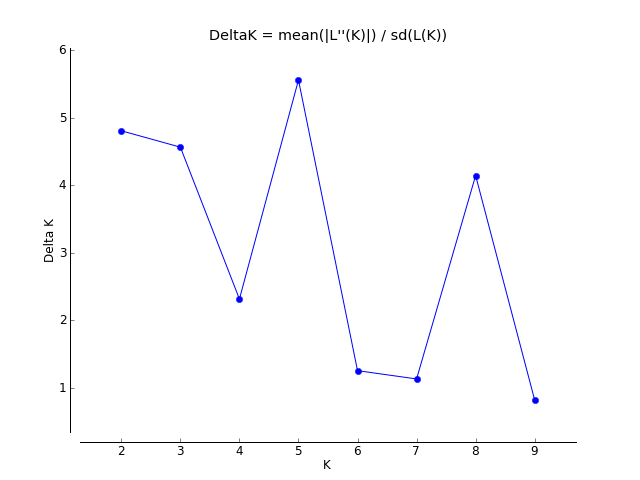


Supplementary Figure 2. ∆ K graph of the accessions of *Capsicum* spp. based on SSR markers

## Supplementary Tables

**Supplementary Table 1.** Performance of Dalle-chilli (*Capsicum annuum*)

| Accessions | Fruit colour | Fruit shape | Fruit wt. (g) | Fruit length (cm) | Fruit dia. (cm) | No of seeds/pod | Leaf length (cm) | Leaf width (cm) | Leaf area (cm^2^) | No of  fruits/  plant | Yield per plant (g) |
| --- | --- | --- | --- | --- | --- | --- | --- | --- | --- | --- | --- |
| SKCC-1 | Greenish Purple | Round | 2.70 | 1.25 | 1.45 | 15.50 | 12.40 | 7.85 | 101.30 | 90.00 | 300.30 |
| SKCC-2 | Green | Round | 2.65 | 1.15 | 1.70 | 26.00 | 12.65 | 8.50 | 49.59 | 80.00 | 261.80 |
| SKCC-3 | Green | Oblong | 2.50 | 2.55 | 1.45 | 24.00 | 9.70 | 5.90 | 84.17 | 69.50 | 192.72 |
| SKCC-4 | Green | Round | 3.10 | 1.35 | 1.70 | 25.00 | 13.40 | 8.50 | 86.28 | 75.50 | 247.50 |
| SKCC-5 | Greenish Purple | Round | 3.75 | 1.45 | 1.55 | 62.00 | 15.15 | 9.40 | 136.90 | 70.50 | 330.00 |
| SKCC-6 | Greenish Purple | Round | 1.90 | 1.15 | 1.45 | 32.50 | 15.85 | 9.85 | 140.50 | 103.50 | 201.96 |
| SKCC-7 | Green | Round | 2.85 | 1.35 | 1.50 | 33.50 | 15.05 | 11.10 | 149.00 | 113.50 | 422.40 |
| SKCC-8 | Greenish Purple | Round | 2.65 | 1.45 | 1.55 | 21.50 | 16.50 | 9.15 | 141.34 | 80.00 | 233.75 |
| SKCC-9 | Green | Round | 2.70 | 1.40 | 1.60 | 26.00 | 16.00 | 9.55 | 143.50 | 70.50 | 214.50 |
| Mean |  |  | 2.76 | 1.46 | 1.55 | 29.56 | 14.08 | 8.87 | 114.73 | 83.67 | 267.21 |
| SD |  |  | 0.49 | 0.43 | 0.10 | 13.31 | 2.22 | 1.45 | 35.42 | 15.67 | 73.52 |
| SE Mean |  |  | 0.16 | 0.14 | 0.03 | 4.44 | 0.74 | 0.48 | 11.81 | 5.22 | 24.51 |

**Supplementary Table 2.** Performance of King-chilli (*Capsicum chinense*)

| Accessions | Fruit colour | Fruit shape | Fruit wt. (g) | Fruit length (cm) | Fruit dia. (cm) | No of seeds/pod | Leaf length (cm) | Leaf width (cm) | Leaf area (cm^2^) | No of  fruits/  plant | Yield per plant (g) |
| --- | --- | --- | --- | --- | --- | --- | --- | --- | --- | --- | --- |
| King Chilli-1 | Red | Long | 5.65 | 8.55 | 3.55 | 36.00 | 17.20 | 9.15 | 170.05 | 45.50 | 278.30 |
| King Chilli-2 | Pale yellow | Long | 3.65 | 3.25 | 2.80 | 39.00 | 11.50 | 5.15 | 77.19 | 33.50 | 146.30 |
| King Chilli-3 | Purple | Long | 4.25 | 5.55 | 3.25 | 35.50 | 14.87 | 6.75 | 125.50 | 29.00 | 132.00 |
| King Chilli-4 | Red | Long | 6.25 | 7.25 | 2.45 | 38.50 | 16.99 | 7.40 | 149.52 | 29.00 | 214.50 |
| King Chilli-5 | Red | long | 3.75 | 4.00 | 2.70 | 33.00 | 13.20 | 5.80 | 100.58 | 28.00 | 118.80 |
| Mean |  |  | 4.71 | 5.72 | 2.95 | 36.40 | 14.75 | 6.85 | 124.57 | 33.00 | 177.98 |
| SD |  |  | 1.17 | 2.20 | 0.44 | 2.43 | 2.45 | 1.55 | 37.12 | 7.31 | 67.12 |
| SE Mean |  |  | 0.52 | 0.99 | 0.20 | 1.09 | 1.09 | 0.69 | 16.60 | 3.27 | 30.01 |

**Supplementary Table 3.** Performance of Cherry chilli (*Capsicum annuum*)

| Accessions | Fruit colour | Fruit shape | Fruit wt. (g) | Fruit length (cm) | Fruit dia. (cm) | No of seeds/pod | Leaf length (cm) | Leaf width (cm) | Leaf area (cm^2^) | No of  fruits/  plant | Yield per plant (g) |
| --- | --- | --- | --- | --- | --- | --- | --- | --- | --- | --- | --- |
| MLCC-35 | Green | Round | 0.28 | 0.65 | 0.85 | 25.00 | 9.80 | 8.50 | 54.50 | 208.50 | 74.25 |
| MLCC-34 | Purple green | Round | 0.70 | 0.85 | 0.90 | 37.00 | 14.75 | 8.70 | 138.50 | 192.50 | 115.50 |
| Mean |  |  | 0.49 | 0.75 | 0.88 | 31.00 | 12.28 | 8.60 | 96.50 | 200.50 | 94.88 |

**Supplementary Table 4.** Performance of Bird’s eye chilli (*Capsicum fruetscens*)

| Accessions | Fruit colour | Fruit shape | Fruit wt. (g) | Fruit length (cm) | Fruit dia. (cm) | No of seeds/pod | Leaf length (cm) | Leaf width (cm) | Leaf area (cm^2^) | No of  fruits/  plant | Yield per plant (g) |
| --- | --- | --- | --- | --- | --- | --- | --- | --- | --- | --- | --- |
| MZBEC-1 | White | Long | 0.95 | 2.90 | 0.60 | 6.00 | 15.30 | 9.45 | 140.25 | 115.50 | 108.90 |
| MZBEC-2 | Green | Long | 0.89 | 2.55 | 0.45 | 13.50 | 10.90 | 6.45 | 87.62 | 76.00 | 78.77 |
| MZBEC-3 | Green | Small Long | 0.40 | 1.70 | 0.55 | 5.50 | 15.40 | 7.25 | 126.90 | 77.50 | 25.41 |
| MZBEC-4 | Green | Small Long | 0.36 | 0.90 | 0.45 | 5.00 | 11.85 | 6.45 | 87.16 | 86.50 | 30.98 |
| MZBEC-5 | Dark Green | Mid bold | 0.29 | 0.70 | 0.35 | 4.50 | 13.50 | 7.05 | 139.25 | 70.50 | 24.75 |
| MZBEC-6 | Green | Mid Long | 0.69 | 2.40 | 0.55 | 11.00 | 9.65 | 5.35 | 45.92 | 102.50 | 68.20 |
| MZBEC-7 | Green | Long | 0.45 | 1.60 | 0.55 | 11.00 | 10.25 | 5.25 | 75.06 | 70.50 | 33.00 |
| MZBEC-8 | Green | Small | 0.31 | 0.85 | 0.50 | 5.00 | 12.83 | 6.15 | 76.78 | 50.50 | 18.48 |
| MZBEC-9 | Green | Small | 0.30 | 1.00 | 0.45 | 5.00 | 14.83 | 7.30 | 81.44 | 75.00 | 24.56 |
| MZBEC-12 | Light Green | Long | 0.75 | 1.30 | 1.05 | 7.50 | 13.10 | 6.65 | 116.49 | 52.00 | 42.24 |
| MZBEC-13 | Green | Long | 1.95 | 4.15 | 0.90 | 21.00 | 6.15 | 2.55 | 22.70 | 96.50 | 203.28 |
| MLCC-1 | White | Long | 1.09 | 2.90 | 0.55 | 20.50 | 14.80 | 7.35 | 114.40 | 79.00 | 95.04 |
| MLCC-39 | Green | Small | 0.39 | 1.30 | 0.45 | 29.00 | 10.80 | 5.70 | 80.40 | 135.00 | 63.80 |
| MLCC-2 | Green | Long | 1.05 | 4.10 | 0.70 | 13.50 | 7.65 | 3.25 | 64.48 | 61.50 | 66.55 |
| Mean |  |  | 0.70 | 2.24 | 0.58 | 11.29 | 11.93 | 6.16 | 89.92 | 82.04 | 63.14 |
| SD |  |  | 0.46 | 1.72 | 0.19 | 7.56 | 2.87 | 1.73 | 34.32 | 23.65 | 49.45 |
| SE Mean |  |  | 0.12 | 0.46 | 0.05 | 2.02 | 0.77 | 0.46 | 9.17 | 6.32 | 13.22 |

**Supplementary Table 5.** Performance of Hot pepper (C*apsicum annum*)

| Accessions | Fruit colour | Fruit shape | Fruit wt. (g) | Fruit length (cm) | Fruit dia. (cm) | No of seeds/pod | Leaf length (cm) | Leaf width (cm) | Leaf area (cm^2^) | No of  fruits/  plant | Yield per plant (g) |
| --- | --- | --- | --- | --- | --- | --- | --- | --- | --- | --- | --- |
| MLCC-36 | Greenish Purple | Long | 4.35 | 4.85 | 1.30 | 33.50 | 12.50 | 9.05 | 59.00 | 122.50 | 618.75 |
| MLCC-37 | Purple Green | Long | 4.75 | 6.95 | 1.55 | 44.50 | 12.75 | 8.75 | 70.50 | 70.00 | 371.25 |
| MLCC-38 | Greenish Yellow | Long | 4.35 | 9.20 | 1.35 | 28.50 | 11.00 | 8.25 | 66.00 | 68.00 | 291.06 |
| MNCC-1 | Greenish Purple | Long | 2.60 | 3.80 | 0.95 | 43.50 | 6.00 | 2.45 | 46.12 | 71.00 | 211.75 |
| MNCC-2 | Green | Long | 6.75 | 5.35 | 2.15 | 54.00 | 11.05 | 5.10 | 66.44 | 31.50 | 284.90 |
| MNCC-3 | Green | Bold long | 3.45 | 3.50 | 1.10 | 101.50 | 7.05 | 2.55 | 28.20 | 41.00 | 168.30 |
| MNCC-4 | Green | Long | 3.05 | 9.65 | 0.75 | 35.50 | 6.65 | 2.90 | 51.52 | 82.50 | 280.50 |
| NLCC-1 | Greenish Purple | Long | 3.25 | 5.55 | 1.15 | 31.00 | 8.35 | 3.20 | 29.49 | 60.50 | 239.58 |
| NLCC-2 | Green | Long | 3.35 | 8.00 | 0.90 | 61.00 | 7.85 | 4.50 | 54.69 | 43.00 | 158.40 |
| NLCC-3 | Green | Long | 3.70 | 9.05 | 1.15 | 17.00 | 8.35 | 3.15 | 34.14 | 39.50 | 175.56 |
| NLCC-4 | Green | Long | 3.65 | 10.10 | 0.75 | 36.50 | 8.50 | 3.00 | 35.31 | 119.00 | 423.50 |
| NLCC-5 | Green | Long | 3.63 | 9.40 | 1.10 | 44.00 | 8.45 | 3.15 | 42.05 | 49.50 | 226.88 |
| NLCC-6 | Green | Long | 4.35 | 12.20 | 0.70 | 34.50 | 6.50 | 2.25 | 25.21 | 56.50 | 321.75 |
| NLCC-7 | Green | Long | 4.10 | 6.50 | 0.95 | 49.00 | 8.60 | 3.65 | 44.01 | 46.00 | 207.90 |
| NLCC-8 | Green | Long | 2.85 | 6.35 | 1.05 | 79.50 | 9.70 | 3.55 | 50.43 | 70.50 | 203.28 |
| NLCC-9 | Green | Round | 2.30 | 1.25 | 1.55 | 55.50 | 7.40 | 3.05 | 35.57 | 22.00 | 60.72 |
| NLCC-10 | Green | Bold long | 5.25 | 4.35 | 1.35 | 106.00 | 7.50 | 3.80 | 50.81 | 64.00 | 411.40 |
| ARC-1 | Green | Long | 2.90 | 3.55 | 1.55 | 26.00 | 7.35 | 3.10 | 53.78 | 23.50 | 82.50 |
| ARC-2 | Dark Purple | Round | 2.40 | 1.30 | 1.40 | 58.00 | 8.70 | 3.85 | 53.02 | 77.50 | 189.75 |
| TRCC-1 | Purple | Long | 2.25 | 3.35 | 0.55 | 31.00 | 9.20 | 3.60 | 73.04 | 52.00 | 116.16 |
| TRCC-2 | Green | Long | 2.60 | 6.65 | 0.75 | 70.00 | 10.00 | 4.15 | 90.83 | 116.50 | 365.31 |
| TRCC-3 | Green | Long | 2.15 | 2.40 | 0.55 | 22.50 | 7.45 | 4.65 | 61.48 | 35.50 | 79.20 |
| TRCC-4 | Green | Long | 2.75 | 6.95 | 0.85 | 83.00 | 6.60 | 2.35 | 46.65 | 55.00 | 194.04 |
| TRCC-5 | Purple | Long | 3.05 | 2.25 | 1.30 | 72.50 | 13.05 | 6.60 | 128.46 | 54.00 | 158.40 |
| TRCC-6 | Green | Long | 2.25 | 3.75 | 0.85 | 53.00 | 8.35 | 3.70 | 78.68 | 92.50 | 242.00 |
| TRCC-7 | Green | Long | 3.35 | 3.40 | 1.15 | 88.50 | 12.75 | 5.65 | 125.00 | 115.00 | 385.00 |
| ASCC-1 | Green | Long | 2.25 | 2.90 | 1.00 | 56.00 | 8.25 | 3.40 | 44.47 | 65.00 | 189.75 |
| ASCC-2 | Green | Long | 3.80 | 8.40 | 0.85 | 72.50 | 8.10 | 3.15 | 55.48 | 140.00 | 564.30 |
| ASCC-3 | Green | Long | 2.85 | 6.35 | 1.05 | 35.00 | 9.70 | 3.55 | 50.43 | 71.50 | 209.44 |
| ASCC-4 | Green | Long | 4.75 | 5.55 | 1.00 | 26.00 | 7.40 | 3.05 | 35.57 | 43.00 | 217.80 |
| ASCC-5 | Greenish Purple | Long | 2.70 | 4.90 | 1.15 | 65.50 | 6.50 | 2.50 | 41.73 | 38.50 | 129.36 |
| ASCC-6 | Greenish purple | Long | 3.25 | 7.10 | 0.75 | 53.00 | 7.85 | 3.00 | 41.50 | 76.50 | 272.25 |
| MLCC-3 | Light Green | Long | 3.15 | 5.75 | 1.25 | 66.00 | 7.70 | 2.75 | 55.03 | 63.00 | 239.58 |
| MLCC-4 | Greenish Purple | Long | 3.00 | 6.00 | 0.95 | 57.50 | 7.80 | 2.95 | 40.18 | 115.50 | 310.00 |
| MLCC-5 | Green | Long | 3.05 | 7.00 | 1.15 | 51.00 | 7.15 | 2.95 | 36.10 | 50.50 | 181.50 |
| MLCC-6 | Green | Long | 1.85 | 4.48 | 0.75 | 35.50 | 9.15 | 3.30 | 72.04 | 89.00 | 193.60 |
| MLCC-7 | Purple | Round | 1.90 | 1.40 | 1.55 | 59.00 | 10.70 | 4.50 | 87.13 | 167.50 | 326.70 |
| MLCC-8 | Green | Long | 2.65 | 3.25 | 1.10 | 70.00 | 10.05 | 4.90 | 60.89 | 71.00 | 203.28 |
| MLCC-9 | Green | Bold Long | 3.35 | 1.50 | 1.95 | 49.00 | 11.85 | 3.85 | 62.12 | 44.50 | 180.95 |
| MLCC-10 | Greenish Purple | Long | 3.25 | 6.65 | 1.05 | 67.50 | 7.85 | 2.50 | 35.31 | 69.00 | 246.84 |
| MLCC-11 | Purple | Long | 2.75 | 3.70 | 1.15 | 61.00 | 7.90 | 3.40 | 69.53 | 51.00 | 175.56 |
| MLCC-12 | Greenish Purple | Long | 2.15 | 5.25 | 0.80 | 41.00 | 6.40 | 2.30 | 27.16 | 111.00 | 246.00 |
| MLCC-13 | Greenish Purple | Long | 2.05 | 4.25 | 0.90 | 46.00 | 7.85 | 2.90 | 45.85 | 91.50 | 219.45 |
| MLCC-14 | Greenish Purple | Long | 2.90 | 6.50 | 0.90 | 23.50 | 11.55 | 4.60 | 56.26 | 112.50 | 346.50 |
| MLCC-15 | Green | Long | 5.50 | 7.00 | 1.35 | 33.50 | 6.45 | 2.25 | 44.85 | 65.50 | 363.00 |
| MLCC-16 | Purple | Long | 3.35 | 5.55 | 0.95 | 17.00 | 6.70 | 2.70 | 39.30 | 80.50 | 272.25 |
| MLCC-17 | Green | Long | 2.75 | 6.60 | 0.90 | 44.50 | 7.45 | 3.40 | 44.28 | 98.50 | 314.16 |
| MLCC-18 | Green | Long | 2.40 | 5.50 | 1.25 | 43.50 | 8.35 | 2.80 | 74.88 | 26.00 | 68.31 |
| MLCC-19 | Green | Long | 2.55 | 5.90 | 0.85 | 24.50 | 8.70 | 2.75 | 49.55 | 69.00 | 187.00 |
| MLCC-20 | Green | Long | 2.05 | 6.65 | 0.65 | 31.50 | 8.00 | 3.00 | 38.30 | 80.00 | 187.00 |
| MLCC-21 | Green | Long | 2.75 | 7.20 | 1.05 | 29.00 | 7.85 | 3.15 | 34.80 | 65.50 | 203.28 |
| MLCC-22 | Greenish Purple | long | 3.35 | 8.30 | 0.85 | 64.00 | 7.90 | 3.20 | 42.56 | 50.50 | 211.75 |
| MLCC-23 | Green | Long | 2.45 | 5.80 | 0.80 | 66.00 | 10.05 | 3.75 | 43.26 | 129.00 | 316.50 |
| MLCC-24 | Green | Long | 1.75 | 5.50 | 0.95 | 31.00 | 7.59 | 2.85 | 46.25 | 70.50 | 152.46 |
| MLCC-25 | Greenish Purple | Long | 2.05 | 4.15 | 1.25 | 109.00 | 8.55 | 2.65 | 43.02 | 70.50 | 145.20 |
| MLCC-26 | Purple | Long | 3.55 | 4.90 | 1.05 | 23.50 | 9.70 | 4.00 | 90.54 | 99.00 | 348.48 |
| MLCC-27 | Green | Long | 3.25 | 5.85 | 1.15 | 13.50 | 8.05 | 3.15 | 36.42 | 70.50 | 264.00 |
| MLCC-28 | Green | Long | 2.65 | 6.60 | 0.85 | 11.00 | 9.15 | 2.70 | 59.10 | 67.00 | 197.34 |
| MLCC-29 | Green | Long | 3.00 | 3.80 | 0.90 | 27.50 | 8.55 | 6.25 | 50.20 | 38.50 | 158.40 |
| MLCC-30 | Greenish purple | Long | 1.90 | 5.20 | 0.70 | 37.00 | 6.80 | 2.75 | 37.34 | 88.50 | 187.00 |
| MLCC-31 | Green | Long | 2.50 | 4.90 | 0.90 | 41.00 | 9.45 | 3.95 | 56.87 | 101.50 | 290.40 |
| MLCC-32 | Green | Long | 2.15 | 5.80 | 0.95 | 59.00 | 6.15 | 2.75 | 24.34 | 65.00 | 173.25 |
| MLCC-33 | Green | Long | 2.75 | 5.65 | 1.05 | 61.00 | 8.80 | 3.45 | 43.10 | 116.50 | 378.84 |
| Kasi Anmol | Green | Long | 3.55 | 6.65 | 1.05 | 56.50 | 8.60 | 3.45 | 44.42 | 101.00 | 415.80 |
| Mahalakshmi | Green | long | 4.35 | 13.55 | 1.00 | 21.00 | 8.40 | 2.55 | 30.36 | 61.50 | 336.60 |
| Surajmukhi | Green | Long | 2.90 | 7.75 | 0.90 | 64.00 | 6.65 | 2.75 | 40.34 | 61.00 | 198.00 |
| Japnese Long | Green | Long | 2.55 | 5.55 | 0.70 | 34.50 | 8.40 | 2.55 | 48.99 | 130.50 | 354.64 |
| Arka Lohit | Green | Long | 3.10 | 7.00 | 0.75 | 23.00 | 7.45 | 2.40 | 42.04 | 110.00 | 369.60 |
| Pusa Jwala | Green | Long | 3.10 | 6.95 | 0.90 | 49.00 | 9.40 | 3.65 | 50.25 | 66.50 | 239.36 |
| Utkal Yellow | green | Long | 2.35 | 7.30 | 0.70 | 61.00 | 9.40 | 3.30 | 58.74 | 74.00 | 197.34 |
| Mean |  |  | 3.08 | 5.80 | 1.04 | 48.15 | 8.54 | 3.60 | 51.87 | 74.36 | 249.30 |
| Sd |  |  | 0.92 | 2.35 | 0.30 | 21.68 | 1.65 | 1.40 | 19.75 | 30.25 | 105.12 |
| SE mean |  |  | 0.12 | 0.29 | 0.04 | 2.71 | 0.21 | 0.17 | 2.47 | 3.78 | 13.14 |

**Supplementary Table 6.** Performance of Sweet pepper (C*apsicum annum*)

| Accessions | Fruit colour | Fruit shape | Fruit wt. (g) | Fruit length (cm) | Fruit dia. (cm) | No of seeds/pod | Leaf length (cm) | Leaf width (cm) | Leaf area (cm^2^) | No of  fruits/  plant | Yield per plant (g) |
| --- | --- | --- | --- | --- | --- | --- | --- | --- | --- | --- | --- |
| Capsicum Long | Green | Long | 13.50 | 8.60 | 3.10 | 142.50 | 11.15 | 4.55 | 69.13 | 22.50 | 303.60 |
| Yellow Wonder | Yellow | Bell shape | 63.00 | 6.60 | 4.70 | 83.00 | 11.67 | 4.65 | 88.91 | 11.00 | 660.00 |
| Orobelle | Yellow | Mid Long | 39.00 | 5.10 | 4.35 | 70.50 | 8.60 | 4.40 | 102.59 | 9.00 | 418.00 |
| Nishant | Green | Long | 39.00 | 5.45 | 4.70 | 67.00 | 9.35 | 4.65 | 66.95 | 13.50 | 627.00 |
| California Wonder | Green | Long | 69.00 | 6.70 | 5.15 | 134.50 | 12.85 | 7.25 | 93.17 | 9.00 | 748.00 |
| Capsicum Red Long | Green | Long | 3.70 | 3.60 | 1.45 | 23.50 | 7.35 | 2.60 | 44.58 | 40.00 | 175.56 |
| Mean |  |  | 37.87 | 6.01 | 3.91 | 86.83 | 10.16 | 4.68 | 77.55 | 17.50 | 488.69 |
| SD |  |  | 25.94 | 1.70 | 1.39 | 44.85 | 2.07 | 1.49 | 21.31 | 12.12 | 224.96 |
| SE Mean |  |  | 10.59 | 0.69 | 0.57 | 18.31 | 0.85 | 0.61 | 8.70 | 4.95 | 91.84 |
